# Potentially adaptive SARS-CoV-2 mutations discovered with novel spatiotemporal and explainable AI models

**DOI:** 10.1186/s13059-020-02191-0

**Published:** 2020-12-23

**Authors:** Michael R. Garvin, Erica T. Prates, Mirko Pavicic, Piet Jones, B. Kirtley Amos, Armin Geiger, Manesh B. Shah, Jared Streich, Joao Gabriel Felipe Machado Gazolla, David Kainer, Ashley Cliff, Jonathon Romero, Nathan Keith, James B. Brown, Daniel Jacobson

**Affiliations:** 1 Oak Ridge National Laboratory, Biosciences Division, Oak Ridge, TN USA; 2 The Bredesen Center for Interdisciplinary Research and Graduate Education, University of Tennessee Knoxville, Knoxville, TN USA; 3 Department of Horticulture, N-318 Ag Sciences Center, University of Kentucky, Lexington, KY USA; 4 Lawrence Berkeley National Laboratory, Environmental Genomics & Systems Biology, Berkeley, CA USA; 5 Department of Psychology, University of Tennessee Knoxville, Knoxville, TN USA

**Keywords:** Molecular evolution, Coronavirus, SARS-CoV-2, COVID-19, Local adaptation, Adaptive mutation

## Abstract

**Background:**

A mechanistic understanding of the spread of SARS-CoV-2 and diligent tracking of ongoing mutagenesis are of key importance to plan robust strategies for confining its transmission. Large numbers of available sequences and their dates of transmission provide an unprecedented opportunity to analyze evolutionary adaptation in novel ways. Addition of high-resolution structural information can reveal the functional basis of these processes at the molecular level. Integrated systems biology-directed analyses of these data layers afford valuable insights to build a global understanding of the COVID-19 pandemic.

**Results:**

Here we identify globally distributed haplotypes from 15,789 SARS-CoV-2 genomes and model their success based on their duration, dispersal, and frequency in the host population. Our models identify mutations that are likely compensatory adaptive changes that allowed for rapid expansion of the virus. Functional predictions from structural analyses indicate that, contrary to previous reports, the Asp^614^Gly mutation in the spike glycoprotein (S) likely reduced transmission and the subsequent Pro^323^Leu mutation in the RNA-dependent RNA polymerase led to the precipitous spread of the virus. Our model also suggests that two mutations in the nsp13 helicase allowed for the adaptation of the virus to the Pacific Northwest of the USA. Finally, our explainable artificial intelligence algorithm identified a mutational hotspot in the sequence of S that also displays a signature of positive selection and may have implications for tissue or cell-specific expression of the virus.

**Conclusions:**

These results provide valuable insights for the development of drugs and surveillance strategies to combat the current and future pandemics.

## Background

The SARS-CoV-2 betacoronavirus, which causes COVID-19, is approaching 70 million infections and more than 1.5 million deaths. Disease outcomes vary geographically and are almost certainly linked to differences in local responses. However, if host biological consequences from infection differ due to mutations within the SARS-CoV-2 genome, it could have profound implications for combating the epidemic. For example, mutations in structural proteins that are targeted by the host immune response may impair the effectiveness of vaccines, and mutations in nonstructural proteins may produce strains that are resistant to antivirals. Therefore, there is an urgent need to determine if segregating polymorphisms are functionally important, which can be done by comparative structural and evolutionary analyses.

Several studies related to the mutagenesis of SARS-CoV-2 have been reported [1–4]. However, these investigations were carried out early in the pandemic and are, therefore, based on small sample sizes and limited geographic distribution. Access to large repositories of globally distributed SARS-CoV-2 sequences, such as Global Initiative on Sharing All Influenza Data, enables the confirmation of preliminary results and the ability to test novel hypotheses. A recent study focused on mutations in the spike glycoprotein and gave indications that both positive selection and recombination may be occurring at the molecular level [5, 6].

Here, we combine structural knowledge with evolutionary analyses of 15,789 full-length globally distributed genomes of SARS-CoV-2 and leverage their haploid, mostly non-recombining nature to generate networks that accurately reflect their spatiotemporal distribution. We use a model based on their duration in the population built on sampling dates (half-life), distribution, and occurrence of each variant to determine the relative success of each haplotype and the mutation that likely allows for that. We then map these mutations on structural models to determine their functional significance. Based on our models, we find that a widely distributed mutation in the spike glycoprotein, Asp^614^Gly, likely alters its quaternary structure conformation and dynamics, and it only became pervasive after a subsequent mutation in the RNA-dependent RNA polymerase. Several other informative subclades were identified that may similarly represent compensatory mutations to counteract deleterious changes, including a variant that is prevalent in the Pacific Northwest of the United States and British Columbia, Canada. We also identify a rapidly evolving genomic region of the nucleocapsid protein that is known to be critical for SARS-CoV replication [7]. Finally, we identify a mutational hotspot in the signal peptide sequence of the spike protein using an explainable artificial intelligence (X-AI) approach. These and other sites warrant continued monitoring as the pandemic spreads and as more refined medical and epidemiological information becomes available.

## Results and discussion

### Overview of the approach

The RNA genome of SARS-CoV-2 is enveloped by a lipidic membrane and its structural proteins, namely, spike glycoprotein (S), envelope (E), membrane glycoprotein (M), and nucleocapsid (N). Once infection is established in the host cell, the viral RNA is replicated and translated, producing 16 nonstructural proteins (nsp1-nsp16) and at least seven auxiliary proteins (ORFs). The overall function of most of these proteins for production of virus is well known (Table 1), and the knowledge gained during the 2002–2004 severe acute respiratory syndrome (SARS) outbreak can add refinements given the high similarity between SARS-CoV-2 and SARS-CoV [8]. Meanwhile, unprecedented efforts of the scientific community have been directed to identify the unique biological features of SARS-CoV-2 leading to the worldwide spread of COVID-19.

**Table 1:** The SARS-CoV-2 proteome (NCBI reference genome NC_045512.2)

Gene	Protein length	Position in the genome	Description
nsp1	180	266–805	Interferes with host mRNA translation and processing [9].
nsp2	638	806–2719	Specific function is not known, it may play an auxiliary role to other viral proteins [10, 11].
nsp3	1945	2720–8554	Papain-like protease with phosphatase activity. Performs proteolytic cleavage of the polyproteins, membrane arrangements and [12, 13, 14].
nsp4	500	8555–10054	Involved in membrane rearrangements during viral infection [14].
nsp5	306	10055–10972	3C-like proteinase that cleave the viral polyprotein to produce the active forms of the nonstructural proteins [15, 16, 17, 18].
nsp6	290	10973–11842	Involved in membrane rearrangements during viral infection and autophagy [15].
nsp7	83	11843–12091	Forms an hexadecameric complex with nsp8 that helps in viral RNA replication [19].
nsp8	198	12092–12685	Forms an hexadecameric complex with nsp7 that helps in viral RNA replication [19].
nsp9	113	12686–13024	Binds and protects the viral genome from host degradation during replication [20, 21].
nsp10	139	13025–13441	Interacts with nsp14 and nsp16 to perform 3′–5′ exoribonuclease and 2′-O-methyltransferase activities, respectively [22, 23].
nsp11	13	13442–13480	Short peptide with potential role in RNA synthesis [24].
nsp12	932	13442–16236	RNA-dependent RNA polymerase [25, 26].
nsp13	601	16237–18039	Viral RNA helicase [27].
nsp14	527	18040–19620	3′-to-5′ exonuclease with proofreading activity [28, 29].
nsp15	346	19621–20658	Nidoviral RNA uridylate-specific endoribonuclease (NendoU) [30].
nsp16	298	20659–21552	2′-O-ribose methyltransferase. Involved in capping of viral mRNA to protect it from host degradation [31].
S	1273	21563–25384	Spike glycoprotein. Interacts with human ACE2 to enter target cells [32].
M	222	26523–27191	Membrane glycoprotein. Required for viral particle assembly [33].
N	419	28274–29533	Nucleocapsid protein. Binds viral RNA during viral particle formation [34].
E	75	26245–26472	Envelope protein. Forms ion channels in host ER membranes. Involved in exaggerated immune response [35, 36, 37].
ORF3a	275	25393–26220	Form ion channels in the host membrane. Linked to inflammatory, IFN signaling, innate immunity, apoptosis, and cell cycle regulation [38, 39, 40, 41, 42].
ORF6	61	27202–27387	Viral replication enhancer [43, 44].
ORF7a	121	27394–27759	Prevents virus tethering at the plasma membrane by inactivation BTS-2 protein [45].
ORF7b	43	27756–27887	Integral transmembrane protein. Its function has not been discovered yet [46, 47].
ORF8	121	27894–28259	Virus replication enhancer [48].
ORF9b^*^	97	28284–28580	Expressed from an alternative reading frame in the N gene. Suppresses host antiviral responses by promoting MAVS degradation [49, 50].
ORF10	38	29558–29674	Potential role in hijacking components of the host ubiquitin-proteasome system (UPS) [50].
ORF14^**^	73	28734–28946	Expressed from an alternative reading frame in the N gene. Unknown function.

^*^Annotated by Gordon et al. [50]

^**^Annotated by Wu et al. [51]

Evolutionary analyses to detect adaptive changes in the virus can be a powerful approach to identify functional processes that should be targeted to combat the pandemic. In addition to ready access to large repositories of full-genome sequences for SARS-CoV-2, such as the Global Initiative on Sharing All Influenza Data (GISAID, gisaid.org) [52], high-resolution structures are available for many of the proteins. With these diverse data types, we can integrate molecular evolutionary analyses with structural models to determine if and how the virus is adapting as it spreads. We tackle these questions in three parts: (*i*) First, we identify the amino acid changes based on the nucleotide sequences and detect signatures of positive and negative selection using a model based on their time-space distribution. (*ii*) We then examine these mutations and their potential functional impact using the recently solved and predicted structures of SARS-CoV-2 proteins. (*iii*) Finally, we use an X-AI approach to identify rare mutational hotspots that are occurring globally across the pandemic, one of which is under positive, directional selection based on standard codon-substitution models of molecular evolution.

### SARS-CoV-2 molecular evolution

#### Gene-based sequence analyses

**Fig. 1:**
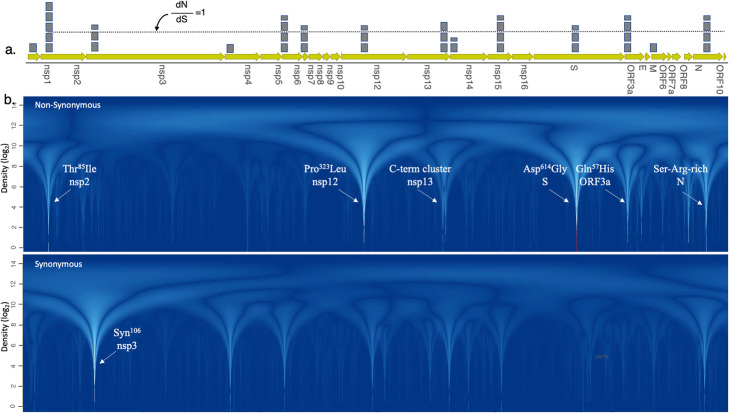
**a**) Ratio of non-synonymous to synonymous mutations (dN/dS) per gene (barplots). We used full-length sequences harboring 395 variable coding sites from GISAID to estimate ratios from the 385 haplotypes detected (see “Methods”). Genes with less than ten mutations across the population and haplotypes with fewer than five individuals were excluded. Ten genes (E, nsp7, nsp8, nsp10, nsp16, ORF6, ORF7A, ORF7B, and ORF8) are likely under high purifying selection at the nucleotide level given that both synonymous and non-synonymous mutations are rare. All changes in a gene were used to calculate dN/dS. Barplots are centered over the strongest signal in a gene. **b**) Wavelet analysis of non-synonymous (top) and synonymous (bottom) mutations across the SARS-CoV-2 genome. Arrows indicate mutation sites discussed in the text. The *y*-axis corresponds to the density of the wavelet across the genome as a log-scale. Higher values indicate a broader wavelet and thus coarser granularity

A simple and common metric to detect selection is the ratio of non-synonymous to synonymous (dN/dS) substitutions among a series of sequences. Under this model, a ratio of one indicates neutral evolution, less than one indicates purifying selection, and greater than one is suggestive of positive selection [53]. We computed dN/dS for the entire genome and then for each gene using the 385 haplotypes identified from the 15,789 full-length sequences of SARS-CoV-2 downloaded from the EpiCov data portal at gisaid.org on June 3, 2020. Given the assumptions of the dN/dS model (i.e., synonymous mutations are neutral), the values indicate that several proteins may be under positive selection with the highest signal in nsp2 (Fig. 1a).

Detection of signatures of selection with the dN/dS ratio is an especially robust and effective approach when comparing sequences that represent significant time intervals since divergence (e.g., sequence variation at the species level). However, the models developed on this metric are built around fixed mutations in each species and not segregating polymorphisms within a species (i.e., population level), and therefore, it can miss selective events [53]*.* Accordingly, as a complementary approach, we used a wavelet analysis to represent the distribution of non-synonymous and synonymous mutations across the SARS-CoV-2 genome (Fig. 1b). The high dN/dS ratio of the N gene from the previous analysis is clearly verified in this analysis and also reveals strong peaks of non-synonymous mutations in nsp2, nsp12, nsp13, and S. Closer inspection reveals that the nsp12 and S signals reflect single mutations found at high frequency within the sampled population (Pro^323^Leu and Asp^614^Gly, respectively), which is also the case with the synonymous mutation at nsp3^106^. The wavelet analysis simultaneously displays the location, frequency, and mutational density at different scales and identified a cluster of moderately frequent mutations involving 14 amino acids in a serine/arginine-rich motif of N (Ser^180^Ile, Ser^183^Tyr, Ser^188^Leu, Ser^190^Asn, Ser^193^Ile, Ser^194^Leu, Arg^195^Lys, Ser^197^Leu, Ser^202^Asn, Arg^203^Lys, Gly^204^Arg, Thr^205^Ile, Ala^208^Val, and Arg^209^Thr) and the C-terminal end of the nsp13 protein.

#### Haplotype success and potential adaptation

**Fig. 2:**
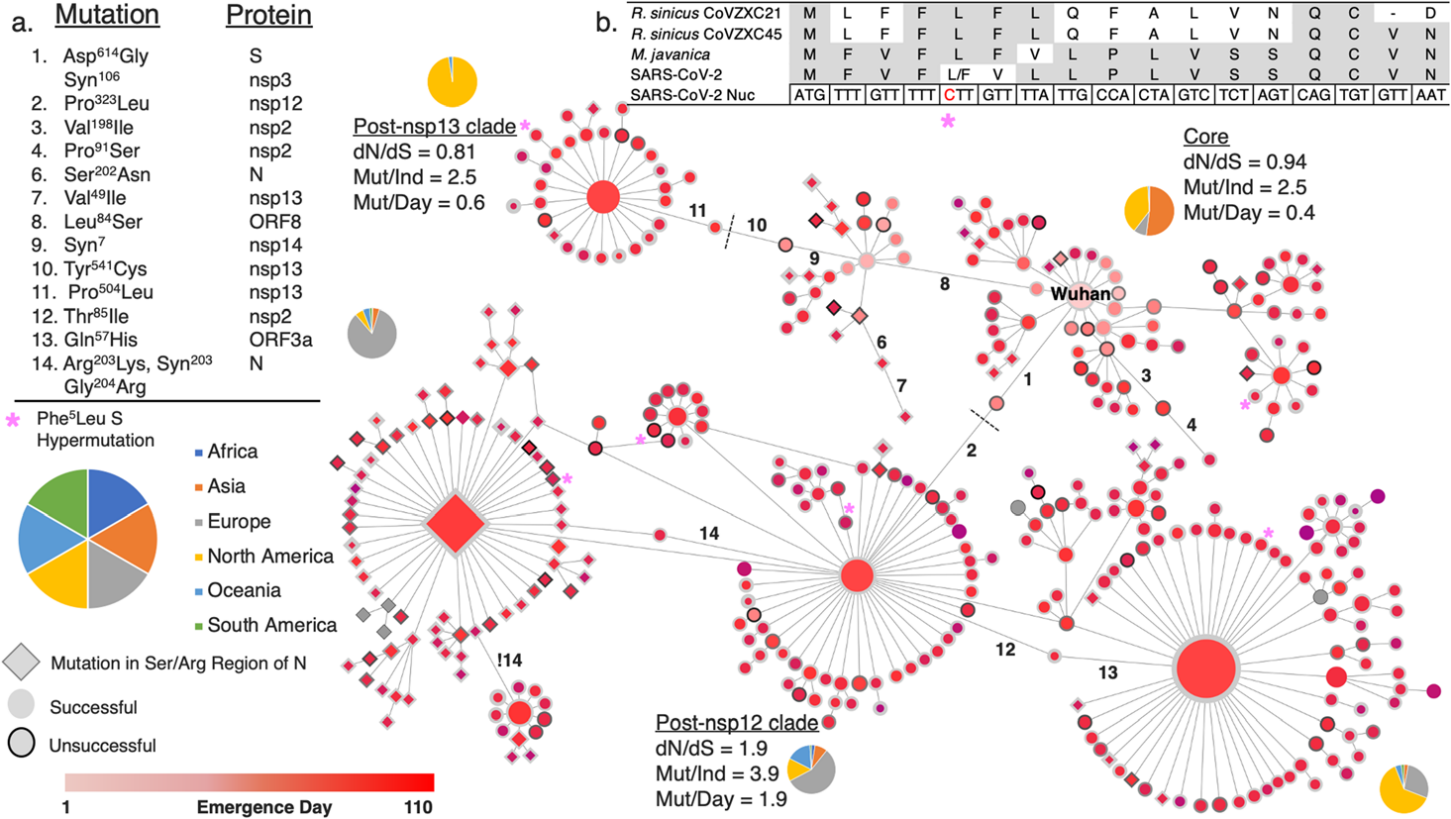
Genealogy and success model of SARS-CoV-2 haplotypes. **a**) Median-joining network of 13,979 full length sequences (haplotypes < 0.05% were removed). Nodes are haplotypes and edges are mutational events. Node size is proportional to the number of individuals. Red gradient in the center of a node indicates the date of emergence (light red haplotype of the Wuhan reference sequence is indicated). Node perimeter darkness reflects the success of a haplotype based on number of days, number of regions, and number of individuals from which it was sampled. Dark perimeter, small diameter nodes indicate haplotypes that persisted globally for long periods but did not expand into many individuals (unsuccessful). Diamonds denote individuals with an amino acid change in the serine/arginine rich region of the N protein (see text). Pie charts indicate geographic distribution of the major nodes. Measures of mutability are given for the three major clades as mutations per day and mutations per individual and dN/dS is provided for each major clade (see text). Exclamation point signifies back mutation to reference sequence. **b**) Alignment of the hyper-mutable region at the signal peptide sequence of S is shown in the upper right. The conserved string of phenylalanine, leucine, and valine residues results in the T-rich region of the signal peptide at the nucleotide level and three runs of the repeat sequence “GTTTT”, which could be responsible for the hyper-mutation. Haplotypes that are linked to individuals with the hypermutable site are shown with a pink asterisk in A (nodes for the haplotypes with hyper-mutation not shown due to low frequency, see “Methods”)

The rapid reporting of full-length genomes and their haploid (and mainly non-recombining) nature allows one to generate what is essentially a mutational genealogy of the virus. A median-joining network can represent the timeline of mutations as the virus spreads across the globe. With that, it is possible to identify mutations that occurred prior to and after a haplotype’s appearance (or removal) from the sampled population. Additionally, knowing the date that a virus was sampled provides a temporal estimate of the half-life of a given haplotype. We define haplotype’s “success” SC (i.e., viral fitness) by the ratio of number of individuals (N) with a given variant to the number of days that variant was sampled (T) and then by the number of geographic regions (G) out of six (Fig. 2) in which it is present [SC = (N/T) × 1/G]. The most successful haplotypes under this model are those that persist for an extended period of time before mutating and they also infect large numbers of individuals across many geographic regions. Particularly, we are interested in viral types that are unsuccessful according to our model, but acquire improved fitness with a subsequent mutational event as these represent potentially adaptive or compensatory responses. These ineffective haplotypes will be those that persist for many days and are found in many geographic regions but at low frequency.

We identified five edges on the haplotype network that may represent deleterious mutations followed by compensatory adaptations that increased its modeled fitness (Fig. 2). Many nodes that appeared to represent unsuccessful haplotypes are found at the tips of the network, and thus, they may have been removed from the population due to purifying selection or severe disease outcome, or they still exist and await further sampling. Here, we focus on five putative compensatory mutations at the internal branches of this haplotype network. The origin of the network corresponds to an unsuccessful haplotype that mutated in a host or hosts as they migrated from China to Europe. This haplotype is defined by the Asp^614^Gly mutation in the S protein (numeral 1, Fig. 2). The next mutation in the virus genealogy (numeral 2, Fig. 2) is a Pro^323^Leu mutation in the RNA-dependent RNA polymerase protein nsp12. A recent paper [54], suggested that the Asp^614^Gly mutation in S allowed the virus to spread to nearly half the global population. Our results, in contrast, indicate that the haplotype that harbors this mutation alone was unsuccessful. However, the subsequent addition of the mutation in nsp12 may have allowed it to spread as a compensatory adaptive change, or possibly Asp^614^Gly is neutral and the nsp12 mutation alone is responsible for the majority of the enhanced viral fitness. Structural analysis indicates that both mutations likely affect the functional performance of these proteins (see “Structural analysis of SARS-CoV-2 mutants” section).

**Fig. 3:**
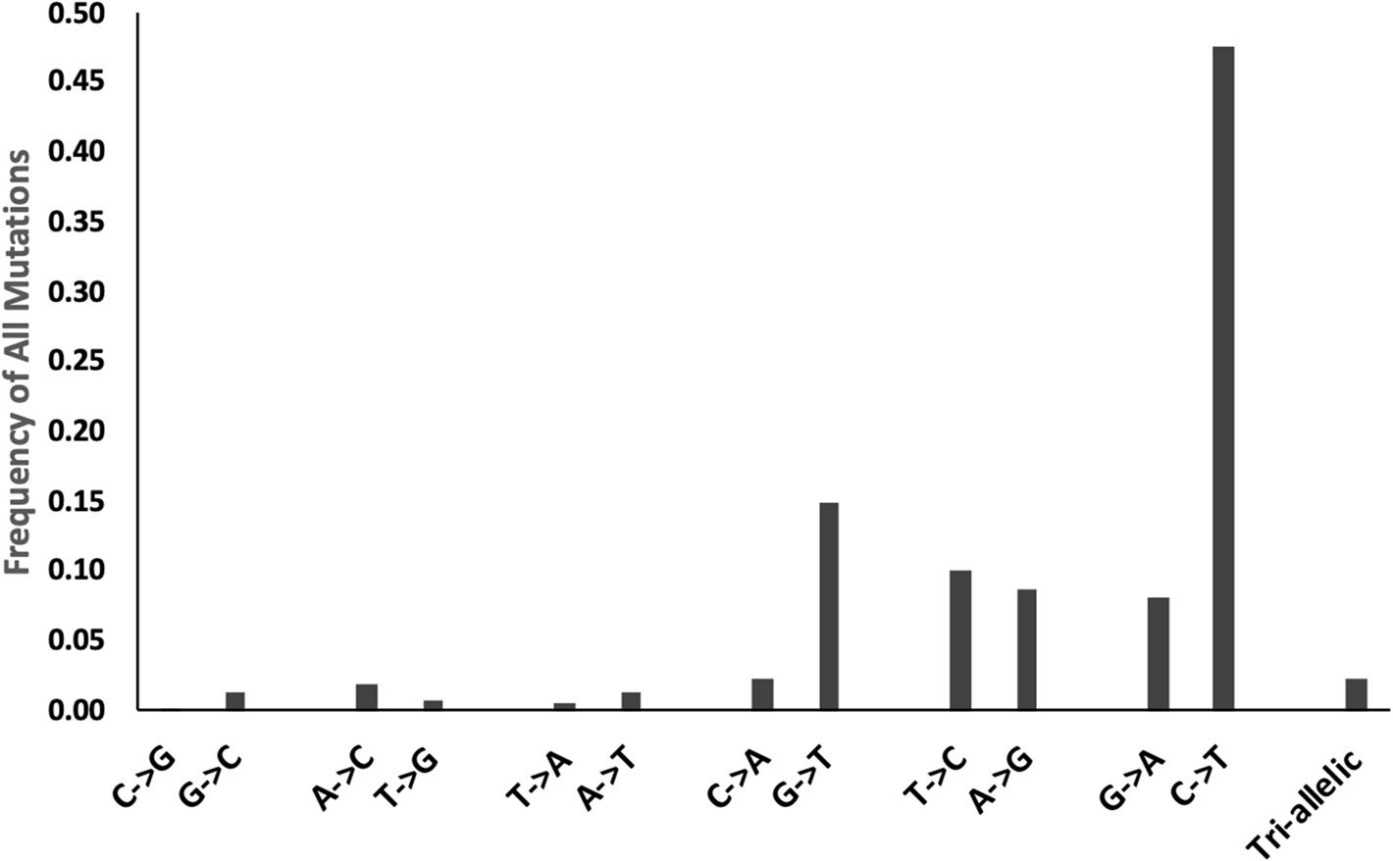
SARS-CoV-2 single-nucleotide mutation spectrum. For each of the twelve classes of mutation, the number of each mutation class at single-mutation variant positions is plotted. The Wu-Hu-1 reference genome (Accession NC_045512.2) was used to define the pre-mutation nucleotide for each class

Interestingly, the large peak in the synonymous wavelet plot (Fig. 1b) corresponds to a mutation in nsp3 at site 106 that is completely linked to Asp^614^Gly in S (i.e., every individual with Asp^614^Gly also carries nsp3^106^). Although synonymous sites are typically considered neutral, when we compared the codon usage of the alternate alleles from SARS-CoV-2 to the pangolin from which may have evolved, there is a significant increase of the codons involved in this mutation (TTT from TTC, *p* < 0.01, Additional file 1: Fig. S1). This suggests that the virus may adapt to hosts by altering its codon preferences, which is supported by the need for codon optimization for efficient expression of coronavirus proteins including those from SARS-CoV-2 in vitro [55–57]. Further, host-mediated deamination, e.g., by ssRNA binding deaminases, may be the major force of evolution in the SARS-CoV-2 genome given that greater than 47% of all identified mutations since the beginning of the pandemic have been C>T transitions—which arise after cytosine deamination (Fig. 3) [58]. Alternatively, it was recently suggested that these changes add or subtract CpG methylation sites as part of an adaptive process by the virus [59].

The next two potentially adaptive events again demonstrate the pattern consistent with our model of compensatory mutation in response to deleterious mutation that rescues the virus (branches 3–4 and 6–7, Fig. 2). In the first instance (branch 3–4), both mutations are in the nsp2 protein. The second event (branch 6–7) consists of the loss of a serine in the N protein at site 202 and a subsequent valine to isoleucine mutation at site 49 in nsp13. It is unclear if there is a compensatory mechanism due to direct contact within these proteins as interactions between them have not been reported. The mutation in N is of particular interest because it is part of the cluster identified in the wavelet analysis (Fig. 1b). Many of the mutations at this serine/arginine-rich region of N do not appear to be successful and are distributed throughout the network (smaller diamond-shaped nodes, Fig. 2). This is consistent with in vitro work in SARS-CoV that determined that mutations at this region decreased virus replication [7].

The fourth potentially adaptive mutational path (branch 9–10–11, Fig. 2) involves two haplotypes found in the state of Washington in the USA that correspond to nearly adjacent mutations in the nsp13 protein (Pro^504^Leu and Tyr^541^Cys). Two recent reports that are still under review stated that no transitional haplotype existed in which individuals harbored only one of these two changes [60, 61]. They hypothesized cryptic transmission events and mutational scenarios to explain the absence of this transitional haplotype. However, we find 11 individuals from nearby British Columbia, Canada, that are infected with a SARS-CoV-2 harboring only the Tyr^541^Cys variant and not the Pro^504^Leu. The short half-life of this haplotype could indicate that the initial Tyr^541^Cys mutation was not sustainable by itself, requiring Pro^504^Leu to persist and expand. Another hypothesis is that the variant transitioned from a host of Asian ancestry to one of non-Asian ancestry in the Pacific Northwest and the second mutation allowed for the adaptation to the new environment, to the genotype of the host, or a combination of the two factors.

The fifth branch (12–13) is defined by the Thr^85^Ile mutation in nsp2 followed by Gln^57^His in ORF3a (Fig. 2) and does not exhibit the low success values as the others but it still may represent an adaptation because there was considerable expansion of the haplotype following it, compared to the previous one from whence it was generated and it also corresponds to two sizeable peaks in the wavelet analysis (Fig. 1b). It may be that our model of haplotype success did not capture it as a compensatory mutation because it appears in a single geographic region rather than many and it was sampled for only a short period of time (2 weeks). In support of it being unsuccessful, it is found in few individuals (only in 11 people from Belgium) and it may be revealed to be of low frequency across different regions as sampling continues.

#### Multiple mutations at a site

From the 395 sites we defined as variable (i.e., found in ten or more individuals), 64 undergo more than one mutation, and 62 of these 64 are in coding regions (Additional file 1: Table S1). At 20 of the 62 sites (32%), the most common alternative allele is a synonymous change. Nearly half (48%) of the second alternate allele are either a synonymous change compared to the reference allele or synonymous compared to the first alternate allele. The majority of multiple mutations at a site are rare, but some are of moderate frequency and regionally specific. For example, an AGC codon (coding a serine) at site 1197 in the nsp3 protein mutated at the third position of AGA (arginine) in 27 individuals from southeast Asia, and in one individual from Australia, beginning in late March. Earlier in March, this same codon mutated to AGT resulting in a synonymous mutation that is currently found mainly in Washington State (14 out of 18 individuals). Notably, 13 sites occur in the N protein, half of which are located in the serine/arginine-rich region, confirming the rapid mutation rate at this functionally important site (Fig. 1).

### Structural analysis of SARS-CoV-2 mutants

Our evolutionary analyses identified several variants that may be important for the adaptation of the virus as it has spread globally. Here, we discuss the potential functional effects that these mutations may have based on structural analyses and, whenever possible, predict how they may alter haplotype performance.

#### Nonstructural protein 2 (nsp2)

It is not yet clear if nsp2 plays a direct role in viral replication. It was, instead, shown that nsp2 binds directly with the host proteins, prohibitin 1 (PHB1) and prohibitin 2 (PHB2), which exhibit a variety of functions in cellular metabolism. Kathiria et al. [62] show that PHB1 knockdown generates reactive oxygen species, mitochondrial depolarization, and induced autophagy. In Hernando-Rodríguez and Artal-Sanz [63], several phenotypes of PHB are reviewed, including the role of PHB in mitochondrial stability. PHB has also been implicated in the inflammatory response in both the lung and gut [63, 64]. In von Brunn et al. [10], it was shown that nsp2 displayed co-immunoprecipitation (CoIP) interaction with other viral proteins, namely nsp3, nsp6, nsp8, nsp11, nsp16, and ORF3a, as well as co-localization with nsp8 and nsp3, where nsp8 almost always co-localized with the microtubule protein, LC3, an autophagy marker protein [11]. As such, it is possible that, by hijacking PHB proteins and perhaps by association with LC3, nsp2 may dysregulate the autophagy defense response or promote mitochondrial dysfunction, therefore enhancing viral replication. The interaction of nsp2 with ORF3a may also trigger mitochondrial dysfunction, as the SARS-CoV protein ORF3a was shown to activate mitochondrial apoptosis [40].

Nsp2 is involved in three potentially adaptive branches. Structural information for nsp2 is scarce, and therefore, we used an *ab initio*-predicted structure to gain preliminary insights of the functional impact identified by the mutations in nsp2 (“Methods”, Additional file 1: Fig. S2). One of the potentially adaptive branches consists of the sequential mutations Val^198^Ile and Pro^91^Ser in nsp2 (branch 3–4, Fig. 2). The first substitution is conservative (properties of valine and isoleucine are similar, Additional file 1: Table S2), and therefore, significant functional impact is not expected. The second mutation, in turn, is predicted to be located at a solvent-exposed loop in the C-terminal domain of nsp2, which potentially affects the interaction of nsp2 with other proteins given that it is located on the protein surface. However, it is too early to infer its effects as it is still a rare event—it is represented by a small terminal node in the haplotype network. The second potentially adaptive branch (branch 12–13, Fig. 2) consists of the mutation Thr^85^Ile at the same loop of nsp2, followed by Gln^57^His in ORF3a; site 57 is predicted to be part of a helix break at the first transmembrane segment. Future biochemical assays, such as CoIP, may determine if these substitutions are compensatory due to the direct interaction between nsp2 and ORF3a, as well as reveal if there is an impact in the interaction with PHB.

#### Nonstructural protein 3 (nsp3)

Nsp3 is a large protein that includes a papain-like protease domain, which is crucial to cleave the viral polyprotein. Although not frequent, several sites in nsp3 underwent more than one mutational event (Additional file 1: Table S1). We identified three different substitutions at site 1198, namely, Thr^1198^Lys, Thr^1198^Ile, and Thr^1198^Arg. This site is located at the surface of a putative nucleic acid-binding domain (residues 1089–1201, Additional file 1: Fig. S3) [65, 66]. We hypothesize that the addition of a positively charged amino acid residue (Lys or Arg) may enhance the electrostatic interaction with the negatively charged regions of the viral RNA genome. Electrophoretic mobility shift assays may be effective to test this hypothesis [67].

#### Replication complex (nsp7, nsp8, and nsp12)

Nsp12 encodes the RNA-dependent RNA polymerase protein, and nsp7 and nsp8 are cofactors, forming together the essential core of the RNA replication complex [68]. Destabilization of this complex was shown to impair its polymerase activity [69].

**Fig. 4:**
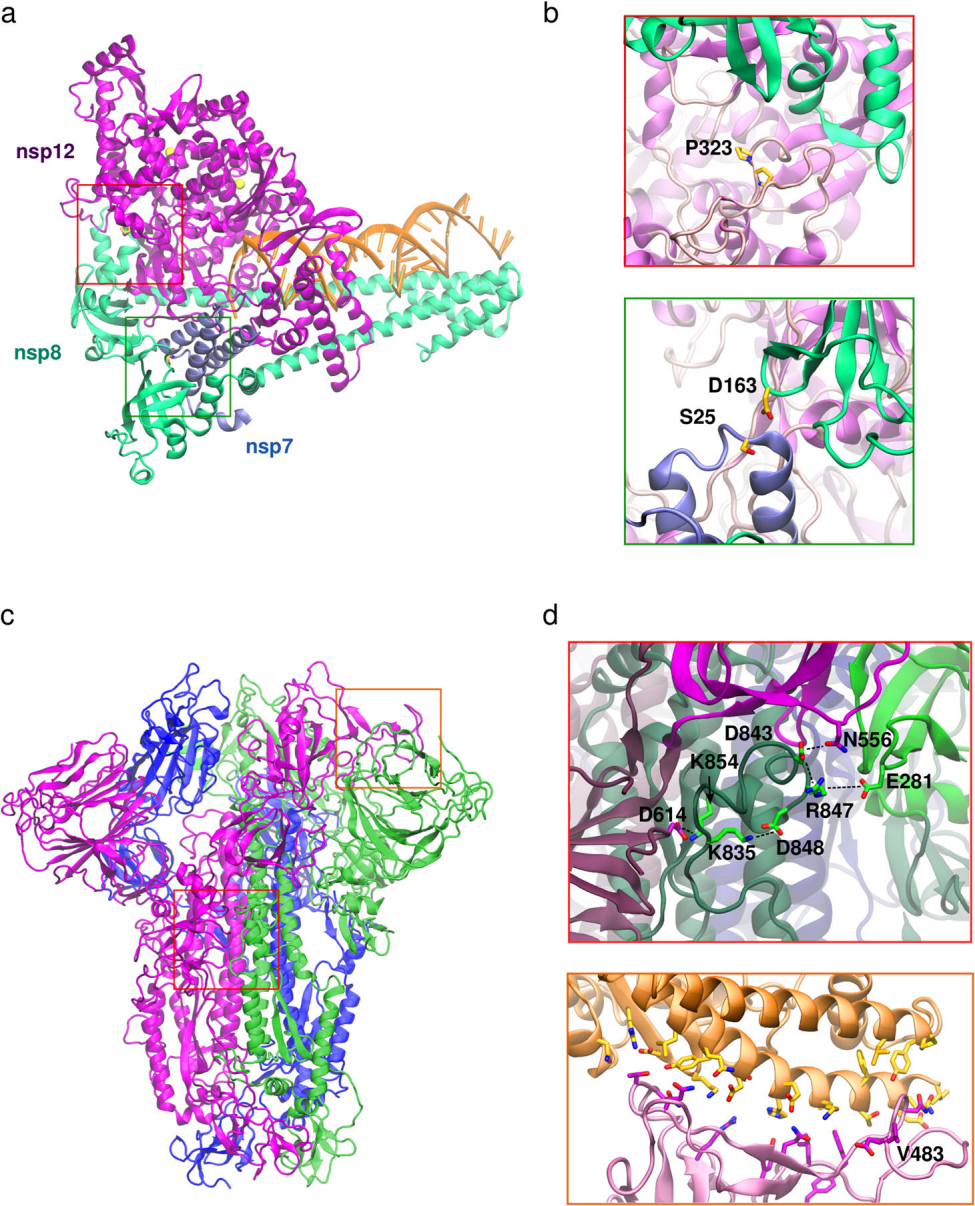
Mutations in the SARS-CoV-2 replication complex (nsp7, nsp8 and nsp12) and spike glycoprotein (S). **a** Active form of RNA-dependent RNA polymerase (nsp12) associated with the cofactors nsp7 and nsp8 (cryo-EM structure, PDB id 6yyt) [26]. **b** View of the proximity surrounding the loop where site 323 of nsp12 is located to nsp8 (red box). Pro^323^Leu is a frequent mutation in nsp12. View of the proximity between Ser^25^ in nsp7 and Asp^163^ in nsp8 (green box), which likely interact with each other via hydrogen bonds. The mutation Ser^25^Leu in nsp7 is fairly frequent. **c** Cryo-EM structure of the S trimer in the closed conformation showing the location of sites 614 (at end of S1 subunit, red square) and 483 (at the β-4,5 loop of the receptor-binding domain, orange square). The cryo-EM structure (PDB id 6vxx) was used in this image. Glycans are not depicted for clarity. The missing loops were modeled using the Rosetta framework [32, 72]. **d** Magnified view of the salt bridge network around Asp^614^ (red box), which may facilitate electrostatic-driven interactions within monomers. The mutation Asp^614^Gly is quite frequent (62% of sequences analyzed have it) (PDB id 6xr8) [73]. Magnified view of the interface between the spike RBD (pink) and ACE2 (orange) (orange box, PDB id 6 m17 [74]). Site 483 is located at the β-4,5 loop of RBD. The mutations Val^483^Gly, Val^483^Ala, and Val^483^Asp were identified in SARS-CoV-2

Several non-synonymous mutations were identified in nsp12, including the Pro^323^Leu, which is one of the mutations in a potentially adaptive lineage in our network (2, Fig. 2) and that has now spread globally (11,338 of the 15,789 sequences from this sample). Using the recently solved cryo-EM structure of the replication complex [26, 70], we verify that it is located in a loop of nsp12 that interacts with nsp8 (Fig. 4a, b). We note that the replacement of a proline in loops is often associated with an increase in local flexibility. Experiments or extensive molecular dynamics simulations can effectively demonstrate the impact of an increased local flexibility in the complex global structure-dynamics. A recent analysis of 220 globally distributed SARS-CoV-2 mutations postulated that this mutation may destabilize the native binding to the cofactors and, thus, affect the proofreading capability of the complex [71]. The study hypothesized that flawed proofreading may be causing an increased mutation rate of the SARS-CoV-2 genome. In order to evaluate that hypothesis, we calculated the mutation rate of our samples in two different ways.

In the first approach, we represent mutation rate as the number of mutations that accumulated per day in the three separate clades (Post-nsp12, Post-nsp13, and Core in Fig. 2) defined by the likely adaptive lineages. In order to only capture the mutations within a clade of interest, we counted mutations from the haplotype that defined it. For the Core clade, the Wuhan reference sequence was used to calculate the number of mutations; for Post-nsp12, the haplotype between numerals 1 and 2 was used; and for Post-nsp13, the haplotype between numerals 10 and 11 was used. We included only the sequences that were sampled for 48 days after the defining haplotype for each clade (see “Methods”). The number of mutations accumulating per day in the viruses carrying the nsp12 mutation is significantly greater than it is for the Core and Post-nsp13 clades (1.9 vs. 0.4 and 0.6, *p* < 6.1 × 10^− 7^), which is consistent with the hypothesis of Pachetti et al. [4]. However, this metric may be biased by how quickly the virus spread or was contained within different geographic regions due to sociodemographic or public health policy. Therefore, as another estimate of mutation rate, we calculated the mean mutation load of the individuals in each of the same three clades. The mean number of mutations per individual in the Post-nsp12 clade is significantly higher than the Core clade or the Post-nsp13 clade (3.9 vs. 2.5, *p* < 1.7 × 10^− 57^), again supporting Pachetti et al. [4]. It is possible that improved fitness could result from a higher mutation rate caused by error-prone replication; the positive trade-off may in fact be an increased replication rate or avoidance of host immune responses by introducing beneficial mutations in other genes [75].

A single Ser^25^Leu mutation is found in nsp7 (250 individuals, mainly in New York and the northeast of the USA) and may also affect replication. Its position in the structure indicates that the serine is a site of hydrogen bond interaction with Asp^163^, in nsp8 (Fig. 4b). Therefore, the substitution to a hydrophobic leucine may affect the native conformation of the replicase complex. Although the mutations in nsp12 and nsp7 are distant from the active site cleft of nsp12, the recently solved structure of the replicase complex indicates that the long helical extensions of nsp8 bind to RNA (Fig. 4a), and therefore, viral transcription itself may be the framework that propagates the local effects of these mutations to substrate binding [26, 70].

#### Nonstructural protein 13 (nsp13)

Potentially relevant mutations were identified within the SARS-CoV-2 helicase (nsp13). The double substitution, Pro^504^Leu and Tyr^541^Cys, defines a large clade that is mostly present in North America (92% of individuals with the mutations). Interestingly, nsp13 is highly conserved among SARS-like coronaviruses [51] and appears to be under neutral or purifying selection as indicated by the dN/dS analysis (Fig. 1a). Both substitutions are located in the 2A (a RecA like) domain of the protein, with Tyr^541^ predicted to be in a region critical for nucleic acid binding (Additional file 1: Fig. S4) [76]. In support of this, a previous in vitro study of SARS-CoV nsp13 showed that the double substitution Ser^539^Ala/Tyr^541^Ala decreased helicase unwinding activity [77]. Based on those results, it is likely that the subtraction of the tyrosine here will impact nucleic acid-binding efficiency, due to the loss of a bulky amino acid that likely interacts with nucleotide bases via π-stacking. The binding region is also associated with the RNA triphosphatase activity of nsp13; thus, the mutation may affect the viral 5′ RNA capping and, thereby, viral replication [27].

The haplotype network shows that this mutation is immediately followed by Pro^504^Leu substitution, which is located at a superficial region of the 2A domain. Our analysis indicates that the *combination* of these two mutations did not significantly affect replication capacity given that the mutational load per individual is only slightly different in the nsp13 clade compared to the core clade (1.6 vs 1.4, *p* < 0.01, Fig. 2). This data suggests that Pro^504^Leu may be compensating the putative loss of performance of Tyr^541^Cys nsp13. The nsp13 protein is known to physically interact with the replicase, nsp12, in SARS-CoV, and the synergistic activity of these proteins enhances nsp13 helicase activity, which is likely important for virus replication [27, 78]. We suggest that future studies should test the hypothesis that Pro^504^Leu directly or indirectly improves nsp12-nsp13 binding and, thus, reestablishes viral replication capacity. This viral lineage has persisted mainly in Washington State and British Columbia (87% of the sequences are found in that part of North America), which suggests local adaptation. However, social distancing may be repressing further distribution.

#### Nucleocapsid

The N protein is assembled and organized in a modular fashion and has been proposed to bind to viral RNA at multiple sites [79]. It self-oligomerizes to encapsulate the viral RNA, and its modular structure is thought to enhance binding affinity with the interacting macromolecules *via* allosteric binding of individual domains. The structured C- and N-terminal domains are linked to each other *via* a long intrinsically disordered region (IDR). IDRs are known to play critical roles in macromolecular interactions, as they confer high inter-domain conformational freedom that allows optimization of favorable contacts [80, 81]. The flexibility and exposure of these protein regions are also associated with a high susceptibility to proteolytic cleavage [82]. IDRs are primary sites of post-translational modifications (PTMs), which can lead to striking changes in protein physicochemical properties. PTMs are often critical to modulate transient folding and/or the assembly of protein complexes mediated by IDRs. Additionally, PTMs that sterically hinder and rigidify the protein backbone are reported to protect IDRs from proteolytic cleavage [83, 84].

In the SARS-CoV-2 N protein, part of the IDR consists of a long serine/arginine-rich segment (Ser/Arg, a.a. 183–206). In vitro experimentation demonstrated that the corresponding motif in the SARS-CoV N protein is crucial for oligomerization [85], and in murine hepatitis virus N protein, this region was shown to be in contact with nsp3 [86]. The nsp3-N interaction is associated with the ability of N to enhance infectivity of coronaviruses [86]. The nucleocapsid is the only structural protein that interacts with the replication/transcription complex [87], and this specific region is directly linked to the replication performance of SARS-CoV [7]. Phosphorylation of the Ser/Arg-rich motif region is reported for SARS-CoV N, and it is suggested to play a role in N antigenicity [88] and nucleocytoplasmic shuttling [89] and, as part of the antiviral immune response, it inhibits the translocation of N to cytoplasmic stress granules [90]. The combination of phosphoryl groups and the guanido moieties of arginines, which can form multiple salt bridges, may explain the importance of this motif to N oligomerization.

The analysis of the GISAID sequences reveals recurrent mutations in the Ser/Arg region of SARS-CoV-2 N protein, reinforcing that this specific region is highly polymorphic in SARS-CoV-2 (Additional file 1: Table S1). Notably, many of these mutations correspond to a loss of potential phosphorylation sites (serines and threonines), namely, Ser^188^Leu, Ser^193^Ile, Ser^194^Leu, Ser^197^Leu, Ser^202^Asn(Ile), and Thr^205^Ile. These mutations do not co-occur concomitantly, which, as discussed above, may avoid disruption of multi-N complexes and, possibly, a significant increase in the susceptibility to proteolysis. Indeed, several of these residues are predicted to be adjacent to a site of proteolytic cleavage (Additional file 1: Fig. S4). Instead, a possible effect of these mutations may be in adding conformational flexibility to the IDR, as well as the addition of sites for hydrophobic interaction. A highly frequent triple mutation is also identified in the Ser/Arg region at sites 28881–28883 of the reference genome (numeral 14, Fig. 2). The impact of this variation, corresponding to a double mutation at the protein level (Arg^203^Lys and Gly^204^Arg), results in an additional positively charged site, and possibly, it increases local rigidity with the subtraction of Gly^204^. Remarkably, this double mutation is the only variation in the Ser/Arg-rich motif that appears at high frequency (Fig. 2), in contrast with the Ser^202^Asn, for example. The bond between Ser^202^ and Arg^203^ is predicted to be a site of proteolytic cleavage by different enzymes (Additional file 1: Fig. S5). Therefore, a preliminary hypothesis to explain the relative success of the double mutation may be that it does not subtract arginines or phosphorylation sites, as other mutations do, avoiding oligomerization disruption and exposure of the protein backbone to proteolysis.

Alternatively, the selection pressure at these sites may be at the transcriptional level. A recent report demonstrated that “AAGAA” motifs in the SARS-CoV-2 transcriptome are enriched in this region and may regulate viral RNAs [91]. One of the rare sites here (28857, Additional file 1: Table S1) removes one of these motifs, and the triple mutation (28881–28883) produces a close match to the motif (“AGGGGAA” to AAACGAA”). This also may explain the low-probability reversion of the 3-bp change in the Ser/Arg-rich region from “AAC” back to “GGG” (!14, Fig. 2). The replicase pauses at these motifs, likely as result of base modification, which could lead to either slip-strand replication, causing increased mutations, or template switching that would effectively result in recombination if another variant was present (co-infection of a host cell).

Finally, the mutation Asp^103^Tyr is within the peak identified in the wavelet analysis. It is located in a protruding β-hairpin in the N-terminal domain of N protein (Additional file 1: Fig. S6). The N-terminal domain serves as the RNA binding site and is rich in exposed aromatic and basic amino acid residues. The abundant aromatic residues are known to interact with RNA bases *via* *π*-stacking and, therefore, the reported mutation may constitute an additional site for RNA recognition.

#### Spike glycoprotein

The binding of the spike glycoprotein to the host receptor, angiotensin-converting enzyme 2 (ACE2), results from a conformational selection mechanism, in which there is a stabilization of the “up” conformation (open state) relative to the “down” conformation (closed state) that exposes or conceals its receptor-binding domain, respectively. As discussed above, within the SARS-CoV-2 GISAID sequences, there is a very frequent (72% of the sampled sequences) Asp to Gly substitution at position 614 at the terminus of the S1 subunit of the spike protein. Despite the relatively low resolution of the solved structures of S in the different states (2.9 Å and 3.5 Å, PDB id: 6xr8 and 6vsb, respectively) [73, 92], two published models indicate that Asp^614^, located in the S1 subunit, is involved in the interaction between subunits and monomers of S (Fig. 4c, d).

In a recent study from the Scripps Research Institute (https://www.scripps.edu/), not yet peer-reviewed, pseudoviruses containing the variant Asp^614^Gly of SARS-CoV-2 S were able to infect HEK293T cells with significantly higher efficiency than those containing the native S protein. Interestingly, ablation of the furin cleavage site of S had a similar effect. This study also suggests a correlation between higher infectivity and decreased S1 shedding, which contradicts a competing hypothesis that this mutation favors infectivity by loosening the intermolecular interactions within the S trimer [54]. Based on the models derived from cryo-EM, it was hypothesized that the mutation results in the loss of a hydrogen bond interaction between Asp^614^ and Thr^859^, which resides in different S monomers [54]. However, the cryo-EM models used to support this hypothesis were generated from a soluble construct of S, which includes two proline stabilizing mutations (PP), which lacks loops near site 614 that may be relevant to better understand the importance of Asp^614^.

The structure of the region surrounding the 614 site has since been determined with cryo-EM using the full-length S by Cai et al. [73]. Visual inspection of this structure shows that Arg^614^ forms a salt bridge with Lys^854^, which is located in the neighboring protomer directly adjacent to a region that Cai et al. designated as the fusion-peptide proximal region (FPPR, residues 828–853). As shown in Fig. 4c, the substitution Asp^614^Gly likely dramatically perturbs the network of salt bridge and hydrogen bond interactions involving residues of the FPPR (Lys^835^, Asp^848^, Arg^847^, and Asp^843^), Asn^556^, at the CTD1, and Glu^281^, at the N-terminal domain (NTD). As CTD1 and NTD are in direct contact with the receptor-binding domain (RBD), this network of interactions with FPPR may be a structural core communicating between residue 614 and the RBD. In fact, by obtaining the first cryo-EM derived map of the Asp^614^Gly S variant, Yurkovetskiy et al. suggest that the mutation at residue 614 leads to a major conformational shift toward “open” states, with more than one receptor-binding domain accessible to interact with ACE2 [93].

In agreement with the Scripps study, Yurkovetskiy et al. also show that the Asp^614^Gly S variant significantly increases the infectivity of pseudotyped lentiviruses in human cell cultures and on cells bearing ACE2 orthologs from other mammals. Notably, despite the higher probability of exposure of the RBDs, the dissociation rate of the Asp^614^Gly S variant from ACE2 is 4-fold faster than the wildtype, showing that the basis of the increased infectivity of Asp^614^Gly S does not rely on a stronger interaction with ACE2 [93]. Therefore, the mechanism by which the mutation at 614 leads to higher infectivity is not fully clear. As it is a multidomain allosteric protein, the involvement of S in cell infection and cell-cell fusion is particularly complex. Further experiments are needed to reconcile the reported higher infectivity, abundance of open states of S, lower affinity to ACE2, and the decreased S1 shedding caused by the Asp^614^Gly S variant.

While the Asp^614^Gly S variant may be increasing infectivity, a higher propensity to exhibit more extreme symptoms in individuals carrying this variant was not clearly verified [54, 94]. However, as we indicated in our mutational analysis above, viruses that harbored only the Asp^614^Gly S mutation were unsuccessful and it was only the addition of the Pro^323^Leu nsp12 mutation that provided for rapid transmission of the virus throughout the globe (branch 1–2, Fig. 2). There are three potential explanations for this observation: (1) the Asp^614^Gly S mutation conferred greater capacity for the virus to enter cells which resulted in more severe medical outcomes in those individuals (or the infected cells), and therefore, the virus was not able to transmit further. The addition of the Pro^323^Leu in nsp12 may impair not only proofreading capability but also its replicase activity, decreasing the severity of disease which allowed for further transmission in asymptomatic or mildly symptomatic individuals. (2) An alternative, contrasting, hypothesis is that the Asp^614^Gly mutation allowed for more efficient host cell entry, but decreased production of virus by the cell and it was only the addition of Pro^323^Leu nsp12 that enhanced the replication efficiency, resulting in increased production of virus. This is supported by Korber et al., who showed that plasma samples from individuals carrying the virus with Asp^614^Gly S and Pro^323^Leu nsp12 have higher counts of viruses than those that carry neither. However, these results cannot determine which mutation is responsible for this observation because the intermediate haplotype with only the Asp^614^Gly S was not tested. (3) It is possible that the Pro^323^Leu nsp12 led to enhanced transmission simply from increased genome diversity by generating a higher number of mutations, which is supported by our analysis above. Such added genome diversity may provide alternative routes to evade host defenses. Further work on specific strains of the virus (comprising solely Asp^614^Gly S or Pro^323^Leu nsp12, and the combination of both) is needed to clarify which, if any, of these hypotheses are correct*.*

Finally, among the other non-conservative mutations registered for SARS-CoV-2 S, the multiple mutations at site 483, Val^483^Gly (Ala, Asp), may require special attention. Although it was verified in only 24 of the samples from Washington State, USA, this mutation may be relevant as it is located at the β-4,5 loop of RBD, which interacts with ACE2 (Fig. 4d). In the crystal structure of the S-ACE2 complex, Val^583^ is not shown to be in contact with ACE2. It also does not establish inter-domain contacts within S in the closed state. However, because it is located in a long loop, i.e., a region of high mobility, we analyzed molecular dynamics simulations of the S-ACE2 complex to verify if Val^584^ eventually interacts with ACE2. The triplicate simulations suggest very low interaction of this residue with ACE2, having been detected for less than 5% of the simulation time. Neighboring residues in the β-4,5 loop, in turn, such as Asn^487^, exhibit persistent interactions with the host receptor during the simulations (89 ± 1% of the simulation time, Additional file 1: Fig. S7). This result suggests that the mutation to alanine will likely not impact the interaction with ACE2, while the mutation to a negatively charged residue (Val^483^Asp) may alter the interfacial contacts. Particularly, the Val^483^Gly substitution may increase conformational flexibility of the loop. How this propagates to affect binding affinity to the host receptor will be the focus of future studies.

### Mutational hotspot in the signal peptide of the S protein

In a different approach to identifying correlated mutations among SARS-CoV-2 sequences, we used an explainable artificial intelligence (X-AI) algorithm, iRF-LOOP [95], in conjunction with Random Intersection Trees (RIT) [96] to analyze the matrix of variable site mutations. The output of iRF-LOOP is a network in which each variable site mutation is given a score for its ability to predict the presence or absence of another variable site mutation given the population in which individuals are vectors of mutations. From the decision trees built during the iRF-LOOP process, RIT produces multiple sets of mutations that co-occur in at least 10% of the decision pathways of the model, effectively identifying potentially interacting mutations. The RIT Score is the number of sets RIT generates for a given model. The larger this value is, the more sets of mutations there are that are predictive of a given target mutation (y-vector). The RIT Score for the target mutation to a T at site 21575 (21575_T) based on the SARS-CoV-2 reference sequence is higher than that for all other mutations at all other sites in the genome (Additional file 1: Table S3), indicating that it was predictable by many different interacting sets of mutations. Further scrutiny of this mutation across the global population revealed that it represents at least 49 *independent* mutational events in 96 individuals distributed across Asia, Oceania, Europe, and North America, and was sampled over the months of February, March, and April. In order to rule out sequencing error, we verified the mutations by remapping reads from FASTQ files deposited in the Sequence Read Archive (“Methods”). These results confirmed the mutation and identified another individual that was heteroplasmic for the mutation (i.e., carries viral sequences with either a T or a C at this position) and another that is heteroplasmic for a G at position 21570. By definition, this site is under positive selection according to substitution models because there are 49 non-synonymous changes at this site and zero synonymous (thus, the dN/dS ratio is infinity).

The hypermutable site at 21575 corresponds to Leu^5^Phe and the heteroplasmy at 21570 to a Val^3^Gly; both are located in the signal peptide (SP) in the N-terminal domain of the S protein. Although we cannot determine the effects on the structure of the protein and if it directly affects function, it has been shown in vitro in SARS-CoV that altering the SP sequence dramatically alters the expression of S and the production of virus in different cell types [97]. From an evolutionary perspective, the local nucleotide sequence may explain some of the hypermutability of the site. There is a conserved leucine and phenylalanine pair at sites 4 and 5 in SARS-CoV-2, in the coronavirus in pangolin (*Manis javanica*) and bat (*Rhinolophus sinicus*) coronaviruses from which it likely evolved (Fig. 2b). With the presence of phenylalanine, leucine, and valine residues on either side of the conserved Phe^4^ and Leu^5^ (at positions 2, 3, 6, and 7), there is a stretch of the repeat “GTTT” that likely increases replication error by the virus replicase (Fig. 2b). We previously showed that these types of repeat elements are responsible for positive selection in mitochondrial DNA because conservation of amino acids at the protein level maintains codons that provide standing repeat elements that generate mutation at adjacent residues [98]. Indeed, the sequence of S of the two most closely related bat coronavirus sequences (CoVZXC21 and CoVZXC45) are 99% identical [99], but one harbors a full codon deletion just after the SP (Fig. 2b).

The replication error at this site may involve a specific host-virus interaction given that the mutational load of these 96 individuals is significantly higher than the rest of the individuals sampled (6.9 vs. 5.3 mutations per person, *p* < 8 × 10^− 17^). It is highly unlikely that these are systematic errors given that the data were produced from 41 different labs in 15 different countries. Another 18 sites displayed significant iRF scores and several included potential repeat nucleotide patterns that could also cause slip-strand replication errors. Notably, two other sites occur in regions of consecutive phenylalanine and leucine repeats whose codons produce long runs of thymines that would cause slip-strand mispairing in the same manner as 21575_T. The second highest scored site has three alleles (Asp^936^Tyr, Asp^936^His in S) which may explain its significant iRF score, i.e., it is predictive of or predicted by more sites than expected because there are more than two alleles. However, in this case, it is not a repeated mutation to the same nucleotide (e.g., C to T in multiple independent events), but rather, it is a mutation event to a third nucleotide state (tri-allelic).

### Concluding remarks

Our Systems Biology approach integrates evolutionary and proteome-wide structural analyses to provide important functional information linked to mutation events in SARS-CoV-2 that can be used to combat the current pandemic. Extensive research has been focused on mutations in the spike glycoprotein that may affect the S-ACE2 interaction. Here, we detail other structurally important variations among the SARS-CoV-2 strains infecting the human population that may explain differences in disease outcomes and geographic distribution. Thus, we highlight the importance of considering concurrent mutations, instead of individual mutations, in order to evaluate their impact in viral transmission rates. Based on the assumptions of our models, our results indicate that the virus is likely undergoing adaptive evolution that is the result of consecutive mutations in S and nsp12, or perhaps solely due to the change in nsp12 as it was transmitted from Wuhan, China, to Europe. We also identify mutations in nsp13 that may affect replication efficiency and are suggestive of local adaptation in haplotypes specific to the Pacific Northwest of the USA. We note that we performed these same analyses with 9294 sequences downloaded in early April and achieved the same results as this recent update (Additional file 1: Fig. S8), indicating they are robust. Additionally, we find significant geographic “functional homoplasy” in the N protein (i.e., repeated mutations in a region rather than in a single site) in a segment that has been directly linked to the replication capacity of SARS-CoV virus [7]. Based on our model, the majority of the mutations in this Ser/Arg-rich region of N do not appear to be very successful (Fig. 2), which indicates that this could be a valuable drug target for non-coding RNA or similar approaches for therapeutics. We also identify several hypermutable sites and potential underlying molecular mechanisms for their occurrence. Continued monitoring of these sites as they expand may identify further informative haplotypes and adaptive events. As with the Ser/Arg region in N, the performance of specific haplotypes may indicate important targets for drug development by revealing the virus’ vulnerabilities and may allow for strain-specific targeting. The hypermutable site in the signal peptide of S should also be monitored given its importance in tropism. The identification of individuals that have multiple strains of the virus and thus carry both the wildtype and mutant alleles suggests that the frequency is higher than the current estimate.

A valuable follow-up to the work presented here will be the integration of the structural- and evolutionary-based information with phenotypic and demographic data, as they become available, in order to build predictive models that can guide diagnostic and surveillance tools for the COVID-19 pandemic. These models can also incorporate climate data, as temperature has been shown to be a dominant evolutionary driving force in other viral species [100]. Moreover, structural analysis was used to provide insights about the potential functional effects of the mutations and point out directions for a collection of future experiments and computational studies (Additional file 1: Table S4). The workflow developed for this study can readily be implemented in future efforts against pathogen outbreaks.

## Methods

### SARS-CoV-2 networks and metrics

We downloaded 37,420 sequences for SARS-CoV-2 in FASTA format from GISAID (gisaid.org) on June 3, 2020, and aligned with our in-house pipeline to the Wu-Hu-1 reference genome (NC_045512.2). The Wuhan reference genome was used for all downstream metrics because it is the official reference and because it was the first sequence reported. Use of an earlier reference genome, should it exist and be reported in the future, will not change our results. As pointed out for human mitochondrial DNA, changing the reference sequence will not alter conclusions of evolutionary analyses [101]. Certainly, the addition of more ancestral sequences will provide further insight to what we have here, but for accurate and efficient reporting of SARS-CoV-2 mutations, the maintenance of the Wuhan genome as the reference is critical. We used the multiple sequence alignment (MSA) tool MAFFT version 7.467 with 16 threads (--thread 16), Splitting Fragments (--addfragments), constant genome length (--keeplength), automatically selecting the most appropriate strategy (--auto), and deactivating the option for memory saving (--nomemsave) to obtain a better performance. The alignment was trimmed in CLC Genomics workbench (version 20), and sequences that were not full length were removed leaving 16,400 sequences.

In order to be conservative and remove potential bias from sequence errors, for downstream analyses we included only sites that were variable in at least 10 individuals from the population we sampled. We then removed individuals with an “N” at the variable sites leaving 15,789 full-length sequences that produced 1675 haplotypes based on 395 variable sites. For the dN/dS and network analyses, we did not randomly choose haplotypes because of potential sampling bias, rather, we removed haplotypes with fewer than 5 individuals to reduce potential sequencing errors leaving 13,979 sequences that defined 385 haplotypes. The dN/dS ratios were calculated from the 385 haplotypes as presence/absence (frequencies of each were ignored). The full 13,979 sequences representing those 385 haplotypes were formatted in NEXUS and assembled with a Median-Joining Network analysis to generate the network [102] visualized in Cytoscape [103]. Geographic location was based on the metadata from the GISAID website.

For the date of emergence, we used the date of the first reported incidence of that haplotype. For the success metric of each haplotype, we divided the number of individuals that carry that linked series of mutations by the number of days it persisted. This number was then divided by the number of geographic regions it was found in based on the six possible listed in the GISAID meta data (Africa, Asia, Europe, North America, Oceania, and South America) (Additional file 1). Because the success metric includes the division by the number of geographic regions in which the haplotype was found and the use of coarse definitions of geography (i.e., continents rather than countries), sampling bias that is known to occur in GISAID is reduced.

For the mutation rate, we first separated sequences from the three major clades into individual matrices. The nsp13 clade was composed of all individuals after the individuals that make up the haplotype between numerals 10 and 11 in Fig. 2. The sequence for this haplotype was also used to count the number of differences that occurred within that clade. Likewise, the Post-nsp12 matrix included only haplotype sequences before the sequences defined by numerals 12 and 13 in Fig. 2 and after the haplotype between numerals 1 and 2, which was used to count differences. The core sequences were those that remained, and the reference sequence from Wuhan was used to calculate numbers of mutations that were different. In order to normalize for time, we used the first 48 days sampled for each of the clades because the Post-nsp13 clade was only sampled for this duration. We should note that the mutational record of the core Wuhan clade is likely upwardly biased because the pandemic began in November and the first reported sequence was from late December. Therefore, the difference between nsp12 and the core as well as nsp13 and the core is conservative.

To check the hypermutation site at 21575, we identified SRA files for five individuals that carry this mutation (SRR11494735, SRR11578105, SRR11578133, SRR11578169, and ERR4080806). These were aligned to the SARS-CoV-2 reference and all individuals were verified for this mutation. The individual sequence from SRR11621805 was heteroplasmic at the site (carried a T and a C), and another (SRR11779993) was heteroplasmic for a G at position 21,570.

### Wavelet analysis for population features and a range of complexities

Genomic scans for patterns of interest are often done with sliding windows of discrete size. Wavelet transforms allow for similar scans but with varying window sizes, simultaneously revealing patterns that occur at different scales such as SNP and gene density across the genome [104, 105].

We used the R statistical programming language 3.5.0 package “wmtsa” [106] and the Ricker wavelet form in the Continuous Wavelet Transform approach (CWT) [107].

$$ W\left(s,\tau \right)=\frac{1}{\sqrt{s}}\int f(t)\psi \times \left(\frac{t-\tau }{s}\right)\  dt $$

The resulting coefficients indicate the quantity of a given feature at differing scales. Here, genomic data was encoded as 1 for non-reference alleles and 0 for reference and invariant sites. Alternate alleles were summed for each site across the population. The corresponding vector was subjected to wavelet analysis and reported as higher or lower than expected after log2 transformation.

### Iterative random forest

We implemented iterative Random Forest Leave-One-Out Prediction (iRF-LOOP) [95] with vectors of genome variants representing the 395 different sites encoded as 468 variants (some sites had multiple possible variants) as features across 15,789 samples, formatted as a matrix. For each of the 468 variants, its corresponding feature vector was removed and used as the target variable for an iterative Random Forest analysis and the remaining 468 features were used to predict it. Together, the results of these models create a matrix of predictive values for each variant site by all other variants. We then mined the paths from the iRF models with Random Intersection Trees (RIT) [89], which efficiently identifies commonly occurring sets (of varying sizes) in a dataset with the use of its own decision trees, to identify the sets of variants that were chosen to split the data in a recurrent pattern, implying that they explicitly worked together to explain the target variant. This suggests that there is an underlying biology connection, represented by these sets mutations, that has an effect on the target mutation.

### Protein modeling and molecular dynamics simulations

Experimentally solved structures were used in our analyses whenever available. For those not available, we used the models released at https://compsysbio.ornl.gov/covid-19/covid-19-structome/, which were predicted with an ensemble workflow of methods [8]. Among those, there are nsp2, the nucleic acid-binding domain of nsp3, and nsp13 structures. Nsp2 was ab initio modeled using the trRosetta workflow [72]. The structures of the C-terminal domain of nsp3 and nsp13, which are 81% and 99% identical to the templates (PDB id 2 k87 and 6jyt, respectively) [108], were modeled with amino acid replacement on the template and local relaxation, using Rosetta remodel [66, 77] and fixbb [109, 110] applications, respectively.

Three independent molecular dynamics simulations of the RBD SARS-CoV-2/ACE2 complex were performed using a cryo-EM structure as starting configuration (PDB id 6m17). The 2020 version of GROMACS was used to run the simulations [111]. CHARMM36 [112] and TIP3P [113] force fields were applied to represent protein and water, respectively. The octahedral simulation box included the protein complex surrounded by a 15-Å layer of water, as well as sodium and chloride ions (~ 0.16 M) to establish the system’s electroneutrality. An initial energy minimization was performed for 5000 steps *via* the steepest descent. Then, a gradual relaxation of the system was conducted in two phases: (i) 6 ns applying positional constraints on non-interfacial α-carbons, and a slow increase in temperature to reach 298.15 K; (ii) 20 ns with positional constraints applied only to the C-terminal domain of ACE2 and five residues in the core of the RBD. (iii) 10 ns triplicate runs were started with reinitialized atomic velocities, using a random seed. The equilibration phase was not considered in the analysis. The production runs were 300 ns. Temperature and pressure were maintained (1.0 atm) using modified velocity rescaling [114] and Berendsen barostat [115], respectively.

## Supplementary information

**Additional file 1.** **Supplementary Figures** (Haplotype Success and Potential Adaptation **Fig. S1**. Codon usage frequency of the SARS-CoV-2 coronavirus, the pangolin from which it likely evolved, and based on the alternative alleles from all currently known mutations. The TTT codon is significantly greater than expected based on pangolin (p < 0.01), suggesting that there is selection for this mutation.) (Structural analysis of SARS-CoV-2 mutants – nsp2 **Fig. S2.** Ab initio predicted structure of the N-terminal domain of nsp2. Sites of non-conservative substitution are depicted in yellow.) (Structural analysis of SARS-CoV-2 mutants – nsp3 **Fig. S3.** Predicted structure of the Arg/Lys-rich nucleic acid-binding domain of nsp3 (a.a. 1089-1201). Non-conservative substitutions are depicted in green. Positively charged residues are also shown, in yellow.) (Structural analysis of SARS-CoV-2 mutants – nsp13 **Fig. S4.** Structure of SARS-CoV-2 nsp13 based on PDB 6jyt. Non-conservative substitutions are depicted in green. Zinc ions are represented as yellow spheres.) (Structural analysis of SARS-CoV-2 mutants – Nucleocapsid **Fig. S5.** Sites of proteolytic cleavage of the Ser/Arg-rich motif of SARS-CoV2 nucleocapsid, predicted by the ExPASy PeptideCutter tool. The proteases, thermolysin (Therm), Arg-C proteinase (ArgC), proteinase K (ProtK), clostripain (Clost), and trypsin (Tryps), are assigned in their putative specific site of cleavage. The site of cleavage between Ser202 and Arg203 is marked with *.) (Structural analysis of SARS-CoV-2 mutants – Nucleocapsid **Fig. S6.** Crystal structure of SARS-CoV-2 nucleocapsid RNA-binding domain (PDB id 6vyo). Non-conservative substitutions are depicted in orange green. Zinc ions are represented as violet spheres.) (Structural analysis of SARS-CoV-2 mutants – Spike glycoprotein **Fig. S7.** Probability density of residues in the receptor-binding domain of SARS-CoV-2 forming contacts with the ACE2. The cutoff distance of 4 Å between any atom in a pair of residues was used. Bars with standard deviation higher than 50% of the probability density are considered transient interactions in the simulations and not included in the plot, except for the contact of residue 483, which is included for reference.) (Concluding remarks **Fig. S8.** Haplotype network produced with 9,294 sequences demonstrating the same relationships with the larger sample size presented in Fig. 2 (main text).) and **Supplementary Tables** (**Table S1.** The 107 sites with more than 2 alleles (more than one mutational event). The most frequent changes are provided first (column four) followed by second or third mutations, if present. The site in the genome is based on the reference sequence NC_0045512. **Table S1 (cont.)**) (**Table S2.** Groups of conservative substitutions considered in this study. Amino acids in brackets are evaluated after structural analysis.) (**Table S3.** Results from iRF-LOOP. Significant loci based on their ability to predict or be predicted by other loci. Three sites (21575_T, 11083_T, and 11074_T) appear to be hypermutation sites that occur as the result of long stretches of thymines due to consecutive runs of phenylalanine, leucine, and valine residues.) (**Table S4.** Summary of the main mutations, possible observable characteristics they may affect, and suggested future experiments to assess that.) (**Supplementary References** 1. Jia Z, Yan L, Ren Z, Wu L, Wang J, Guo J, et al. Delicate structural coordination of the Severe Acute Respiratory Syndrome coronavirus Nsp13 upon ATP hydrolysis. Nucleic Acids Res. 2019;47:6538–50. 2. von Brunn A, Teepe C, Simpson JC, Pepperkok R, Friedel CC, Zimmer R, et al. Analysis of intraviral protein-protein interactions of the SARS coronavirus ORFeome. PLoS One. 2007;2:e459. 3. Neuman BW, Joseph JS, Saikatendu KS, Serrano P, Chatterjee A, Johnson MA, et al. Proteomics analysis unravels the functional repertoire of coronavirus nonstructural protein 3. J Virol. 2008;82:5279–94. 4. Jia Z, Yan L, Ren Z, Wu L, Wang J, Guo J, et al. Delicate structural coordination of the Severe Acute Respiratory Syndrome coronavirus Nsp13 upon ATP hydrolysis. Nucleic Acids Res. 2019;47:6538–50. 5. He R, Dobie F, Ballantine M, Leeson A, Li Y, Bastien N, et al. Analysis of multimerization of the SARS coronavirus nucleocapsid protein. Biochem Biophys Res Commun. 2004;316:476–83.)

**Additional file 2.** Code used to calculate the success metric.

**Additional file 3.** Review history.

## Data Availability

The genome sequences of SARS-CoV-2 are available in the GISAID repository, https://www.gisaid.org/ and the MAFFT software used to align them is freely available for download (https://mafft.cbrc.jp/alignment/software/). Predicted protein models and molecular dynamics simulations are available at https://compsysbio.ornl.gov/covid-19/covid-19-structome/. Experimentally solved protein structures used are available in the Protein Data Bank, https://www.rcsb.org/. The PDB IDs of these structures are as follows: 6vxx, 6xr8, 6vsb, and 6m17 (spike glycoprotein); 6yyt (replication complex); 2 k87 (nsp3); 6jyt (nsp13); and 6vyo (nucleocapsid). The iterative Random Forest code is available at https://github.com/Jromero1208/RangerBasediRF and https://www.osti.gov/biblio/1560795 with DOI:10.11578/dc.20201001.84, under the GNU General Public License. Random Intersection Tree code is available at https://CRAN.R-project.org/package=FSInteract, under the GNU General Public License. The R code used to calculate the success metric is included as Additional file 2.
