# Supplementary material for: Potentially adaptive SARS-CoV-2 mutations discovered with novel spatiotemporal and explainable AI models
Source: Genome Biol. 2020 Dec 23;21:304. doi: 10.1186/s13059-020-02191-0 (PMC7756312; doi:10.1186/s13059-020-02191-0)
Supplement: Supplementary file 1 — Additional file 1. Supplementary Figures (Haplotype Success and Potential Adaptation Fig. S1. Codon usage frequency of the SARS-CoV-2 coronavirus, the pangolin from which it likely evolved, and based on the alternative alleles from all currently known mutations. The TTT codon is significantly greater than expected based on pangolin (p < 0.01), suggesting that there is selection for this mutation.) (Structural analysis of SARS-CoV-2 mutants – nsp2 Fig. S2. Ab initio predicted structure of the N-terminal domain of nsp2. Sites of non-conservative substitution are depicted in yellow.) (Structural analysis of SARS-CoV-2 mutants – nsp3 Fig. S3. Predicted structure of the Arg/Lys-rich nucleic acid-binding domain of nsp3 (a.a. 1089-1201). Non-conservative substitutions are depicted in green. Positively charged residues are also shown, in yellow.) (Structural analysis of SARS-CoV-2 mutants – nsp13 Fig. S4. Structure of SARS-CoV-2 nsp13 based on PDB 6jyt. Non-conservative substitutions are depicted in green. Zinc ions are represented as yellow spheres.) (Structural analysis of SARS-CoV-2 mutants – Nucleocapsid Fig. S5. Sites of proteolytic cleavage of the Ser/Arg-rich motif of SARS-CoV2 nucleocapsid, predicted by the ExPASy PeptideCutter tool. The proteases, thermolysin (Therm), Arg-C proteinase (ArgC), proteinase K (ProtK), clostripain (Clost), and trypsin (Tryps), are assigned in their putative specific site of cleavage. The site of cleavage between Ser202 and Arg203 is marked with *.) (Structural analysis of SARS-CoV-2 mutants – Nucleocapsid Fig. S6. Crystal structure of SARS-CoV-2 nucleocapsid RNA-binding domain (PDB id 6vyo). Non-conservative substitutions are depicted in orange green. Zinc ions are represented as violet spheres.) (Structural analysis of SARS-CoV-2 mutants – Spike glycoprotein Fig. S7. Probability density of residues in the receptor-binding domain of SARS-CoV-2 forming contacts with the ACE2. The cutoff distance of 4 Å between any atom in a pair of resid [file 13059_2020_2191_MOESM1_ESM.docx]

# Supplementary Figures

*Haplotype Success and Potential Adaptation*


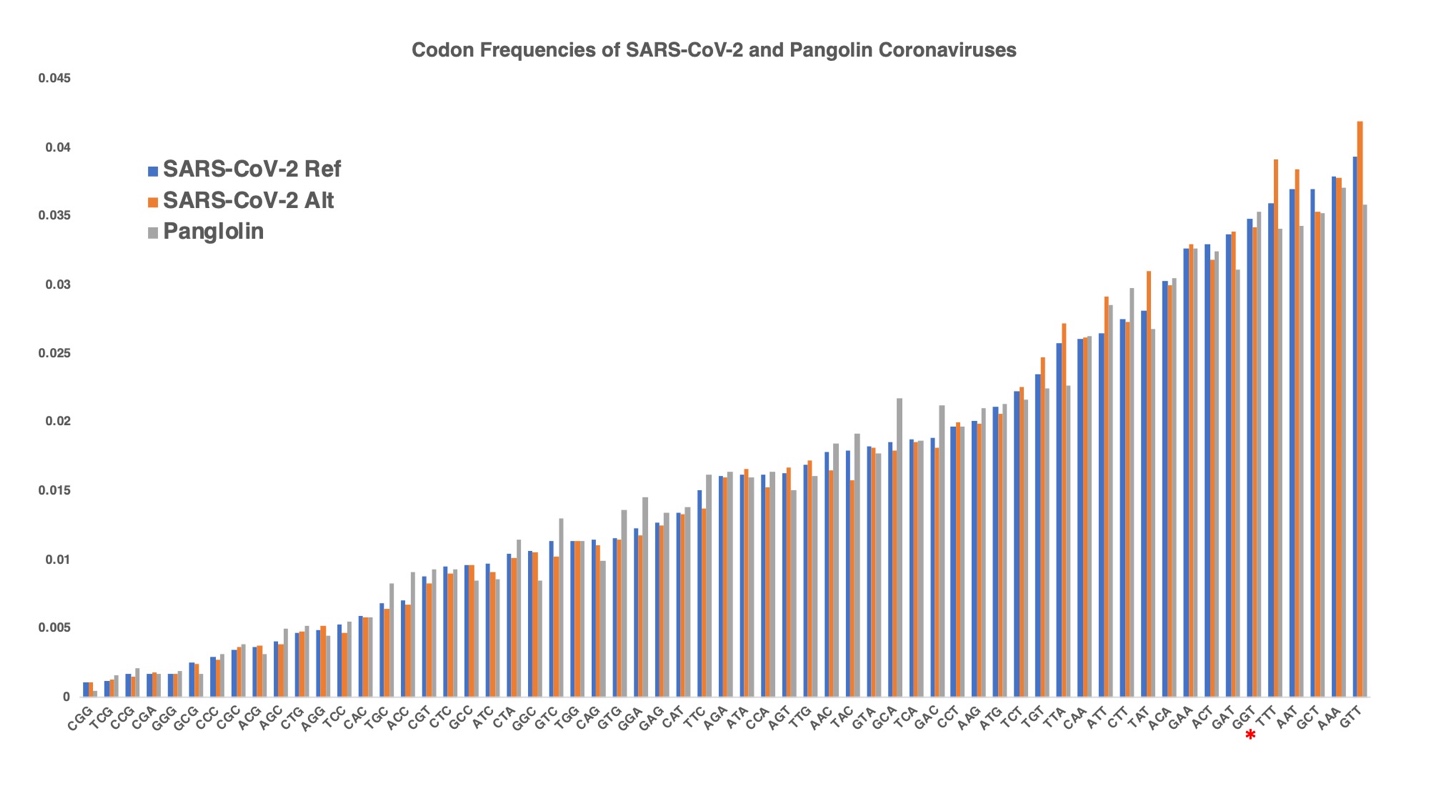


**Fig. S1**.

Codon usage frequency of the SARS-CoV-2 coronavirus, the pangolin from which it likely evolved, and based on the alternative alleles from all currently known mutations.  The TTT codon is significantly greater than expected based on pangolin (p < 0.01), suggesting that there is selection for this mutation.

*Structural analysis of SARS-CoV-2 mutants – nsp2*

**
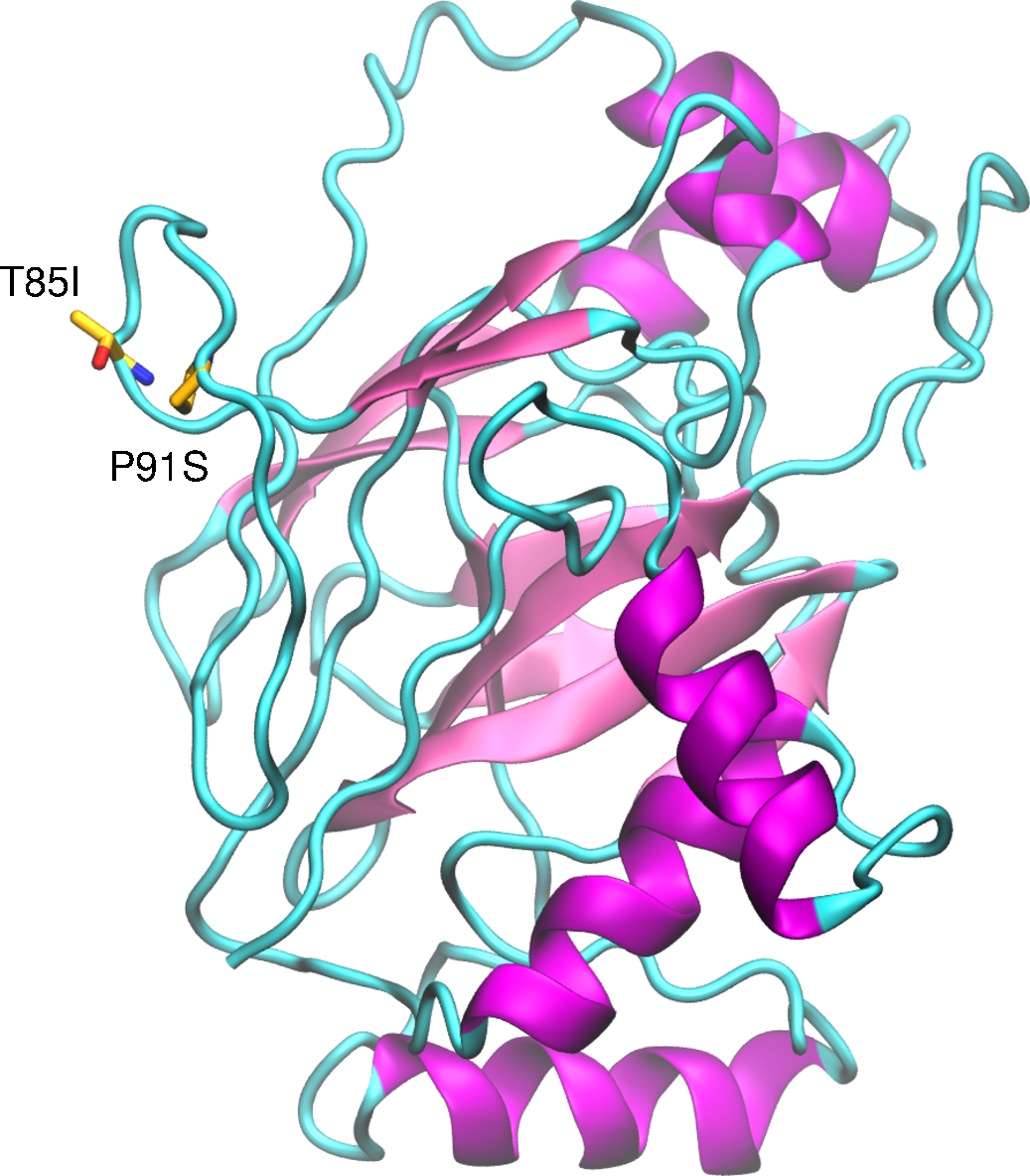
**

**Fig. S2.**

*Ab initio* predicted structure of the N-terminal domain of nsp2. Sites of non-conservative substitution are depicted in *yellow*.

*Structural analysis of SARS-CoV-2 mutants – nsp3*


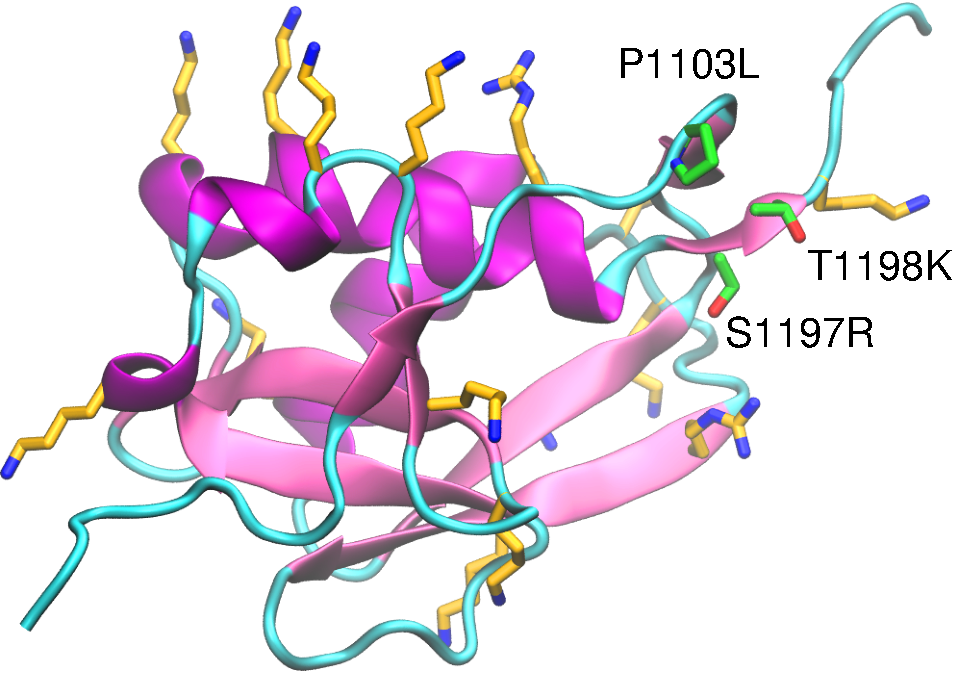


**Fig. S3.**

Predicted structure of the Arg/Lys-rich nucleic acid-binding domain of nsp3 (a.a. 1089-1201). Non-conservative substitutions are depicted in *green*. Positively charged residues are also shown, in *yellow*.

*Structural analysis of SARS-CoV-2 mutants – nsp13*


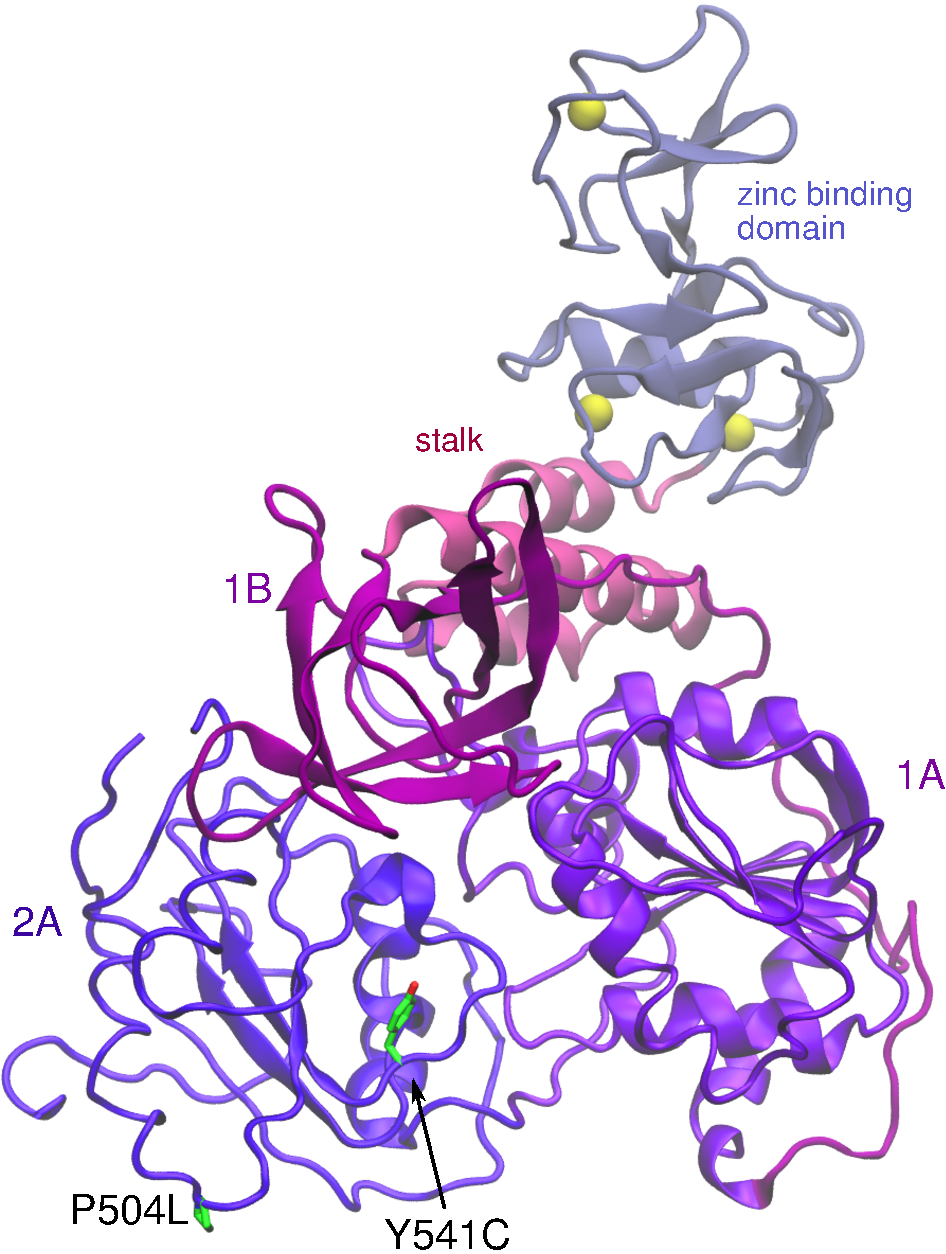


**Fig. S4**.

Structure of SARS-CoV-2 nsp13 based on PDB 6jyt. Non-conservative substitutions are depicted in *green*. Zinc ions are represented as *yellow* spheres.

*Structural analysis of SARS-CoV-2 mutants – Nucleocapsid*

**
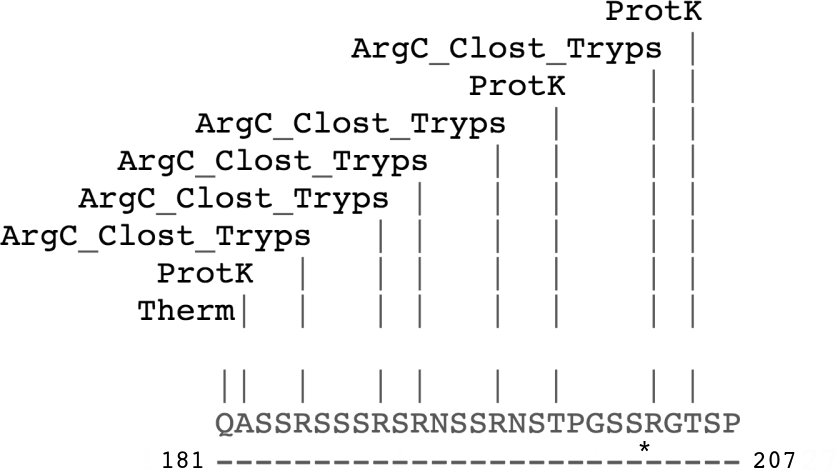
**

**Fig. S5**.

Sites of proteolytic cleavage of the Ser/Arg-rich motif of SARS-CoV2 nucleocapsid, predicted by the ExPASy PeptideCutter tool. The proteases, thermolysin (Therm), Arg-C proteinase (ArgC), proteinase K (ProtK), clostripain (Clost), and trypsin (Tryps), are assigned in their putative specific site of cleavage. The site of cleavage between Ser^202^ and Arg^203^ is marked with *.

*Structural analysis of SARS-CoV-2 mutants – Nucleocapsid*


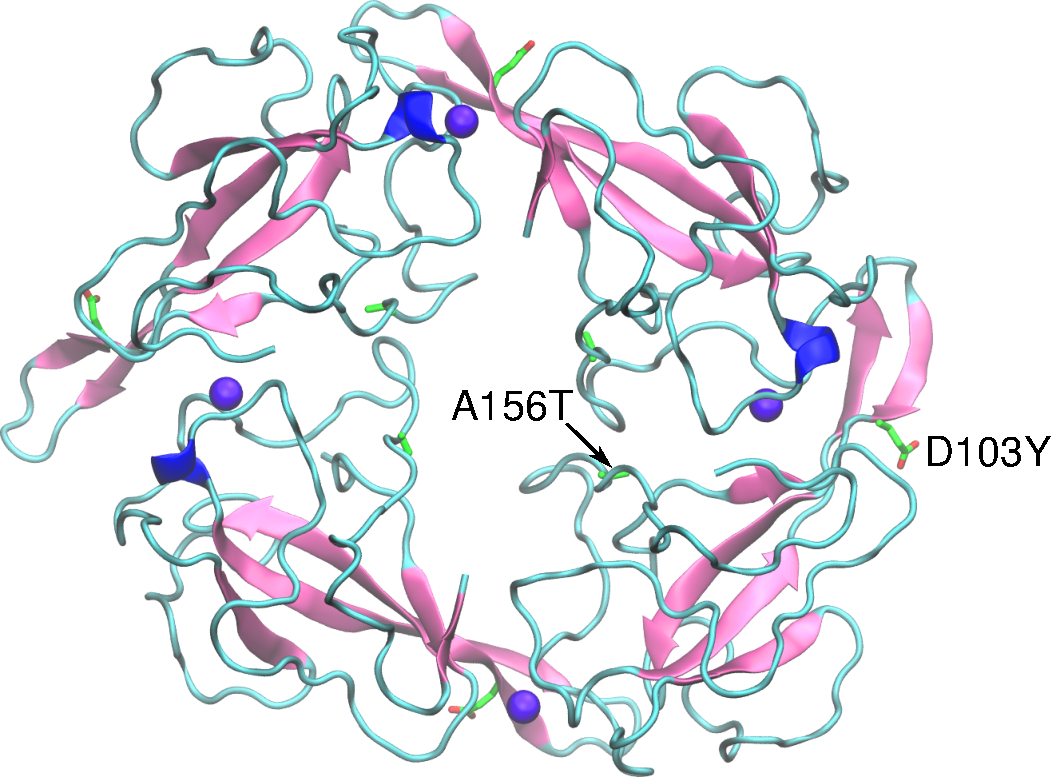


**Fig.  S6.**

Crystal structure of SARS-CoV-2 nucleocapsid RNA-binding domain (PDB id 6vyo). Non-conservative substitutions are depicted in *orange green*. Zinc ions are represented as *violet* spheres.

*Structural analysis of SARS-CoV-2 mutants – Spike glycoprotein*


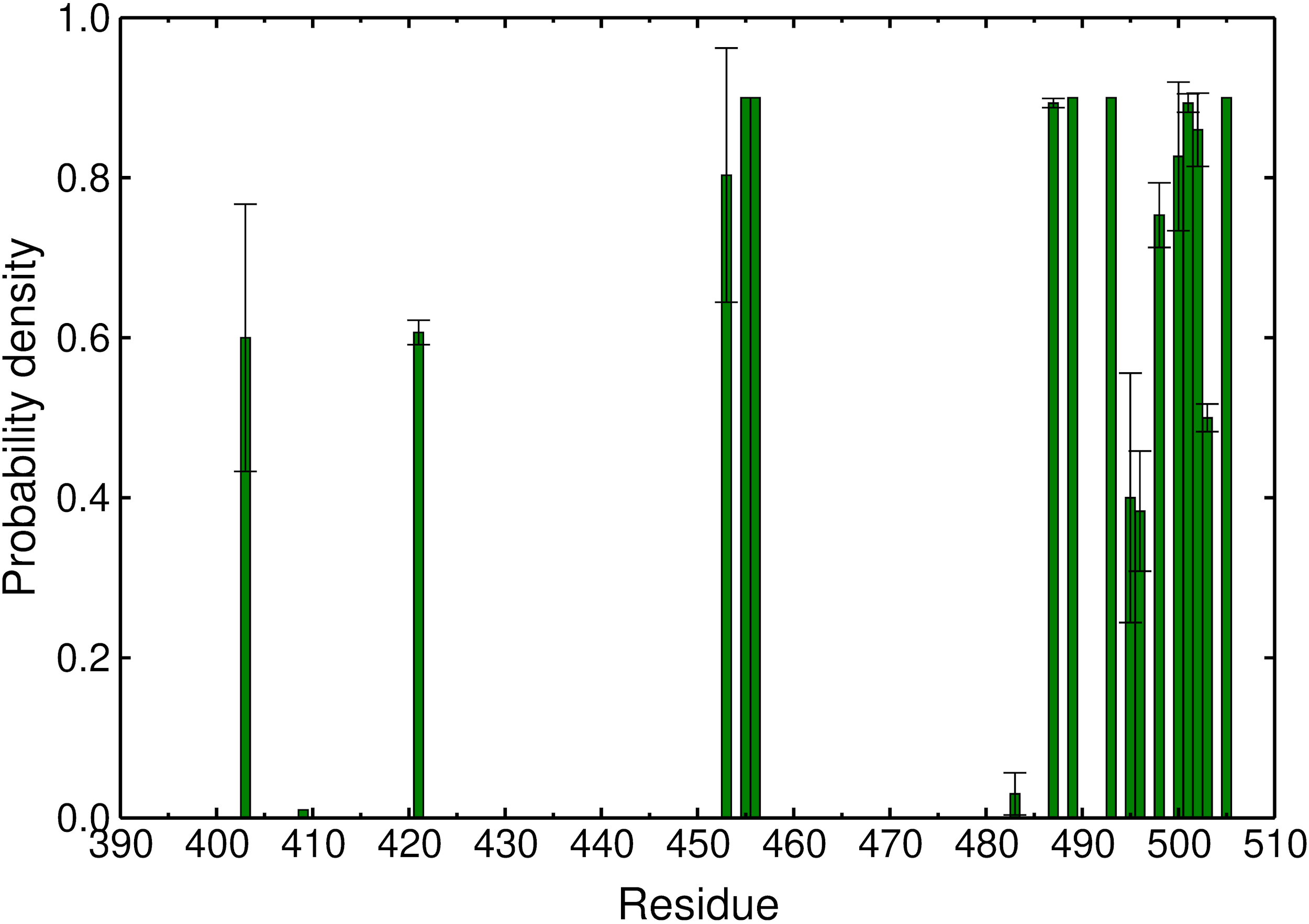


**Fig. S7**.

Probability density of residues in the receptor-binding domain of SARS-CoV-2 forming contacts with the ACE2. The cutoff distance of 4 Å between any atom in a pair of residues was used. Bars with standard deviation higher than 50% of the probability density are considered transient interactions in the simulations and not included in the plot, except for the contact of residue 483, which is included for reference.

*Concluding remarks*


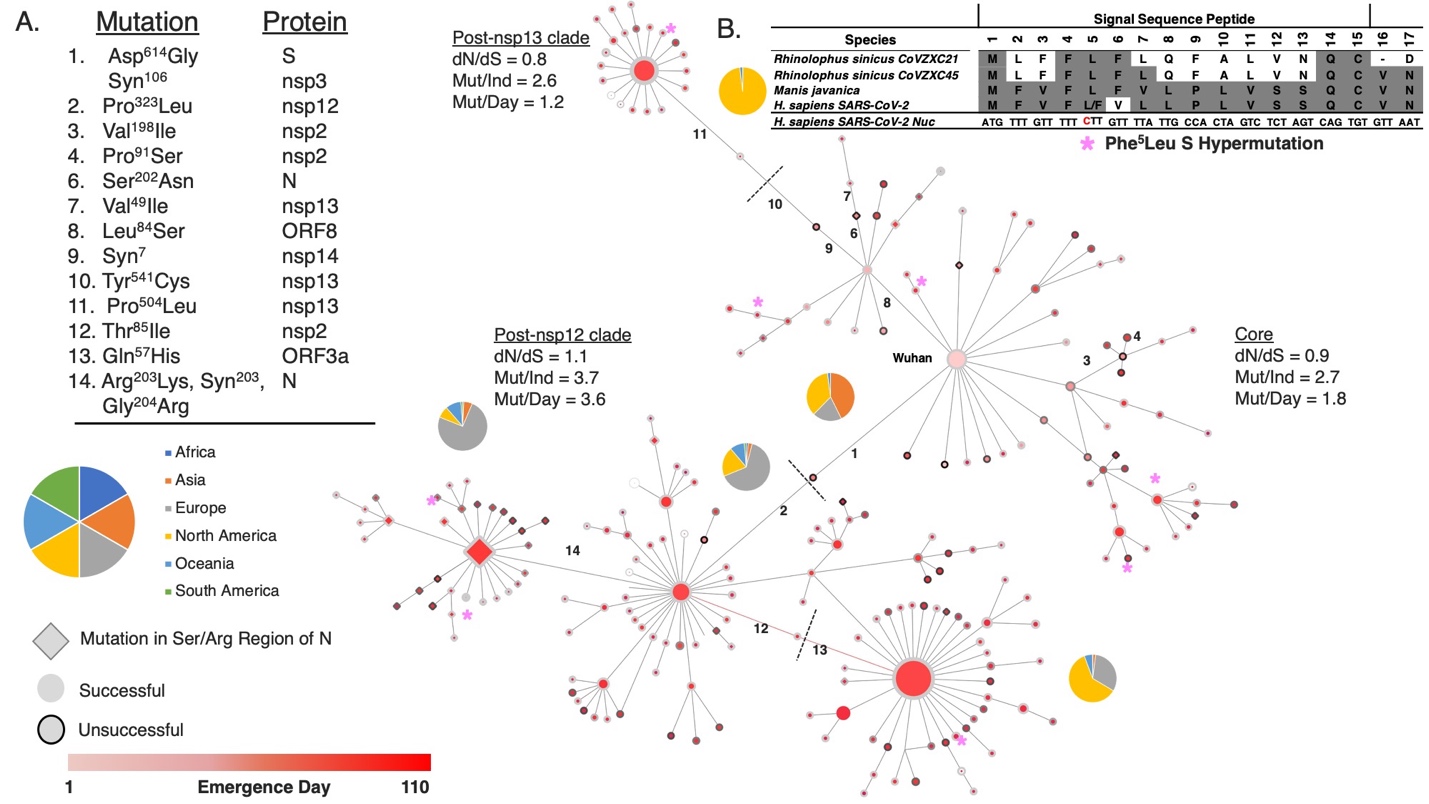


**Fig. S8.**

Haplotype network produced with 9,294 sequences demonstrating the same relationships with the larger sample size presented in Fig. 2 (main text).

# Supplementary Tables

**Table S1.**

The 107 sites with more than 2 alleles (more than one mutational event).  The most frequent changes are provided first (column four) followed by second or third mutations, if present.  The site in the genome is based on the reference sequence NC_0045512.

| **Site** |  | **Mut01** |  | **Mut02** |  | **Mut03** |  | **Gene** |  | **Ref** |  | **A** |  | | **G** | |  | | **T** | |  | | **C** | |
| --- | --- | --- | --- | --- | --- | --- | --- | --- | --- | --- | --- | --- | --- | --- | --- | --- | --- | --- | --- | --- | --- | --- | --- | --- |
| 1440 |  | Gly212Asp |  | Gly212Val |  |  |  | nsp2 |  | G |  | 199 |  | | 15588 | |  | | 2 | |  | | 0 | |
| 1457 |  | Arg218Cys |  | Arg218Met |  |  |  | nsp2 |  | C |  | 2 |  | | 0 | |  | | 20 | |  | | 15767 | |
| 1594 |  | Syn |  | Syn |  |  |  | nsp2 |  | C |  | 2 |  | | 0 | |  | | 38 | |  | | 15749 | |
| 3037 |  | Syn |  | Leu |  |  |  | nsp3 |  | C |  | 1 |  | | 0 | |  | | 11322 | |  | | 4466 | |
| 3177 |  | Pro153Leu |  | Pro153His |  |  |  | nsp3 |  | C |  | 2 |  | | 0 | |  | | 167 | |  | | 15620 | |
| 3373 |  | Asp218Glu |  | Syn |  |  |  | nsp3 |  | C |  | 49 |  | | 0 | |  | | 1 | |  | | 15739 | |
| 4255 |  | Syn |  | Syn |  |  |  | nsp3 |  | G |  | 4 |  | | 15737 | |  | | 48 | |  | | 0 | |
| 5062 |  | Leu781Phe |  | Syn |  |  |  | nsp3 |  | G |  | 3 |  | | 15764 | |  | | 22 | |  | | 0 | |
| 6310 |  | Syn |  | Ile |  |  |  | nsp3 |  | C |  | 45 |  | | 0 | |  | | 18 | |  | | 15726 | |
| 6312 |  | Thr1198Lys |  | Thr1198Ile |  | Thr1198Arg |  | nsp3 |  | C |  | 167 |  | | 2 | |  | | 3 | |  | | 15617 | |
| 7765 |  | Syn |  | Syn |  |  |  | nsp3 |  | C |  | 1 |  | | 0 | |  | | 62 | |  | | 15726 | |
| 8293 |  | Syn |  | Syn |  |  |  | nsp3 |  | C |  | 3 |  | | 0 | |  | | 20 | |  | | 15766 | |
| 9534 |  | Thr327Asn |  | Thr327Ile |  |  |  | nsp4 |  | C |  | 15 |  | | 0 | |  | | 2 | |  | | 15772 | |
| 10798 |  | Asp248Glu |  | Asp248Asp |  |  |  | nsp5 |  | C |  | 96 |  | | 0 | |  | | 2 | |  | | 15691 | |
| 11083 |  | Leu37Phe |  | Syn |  |  |  | nsp6 |  | G |  | 2 |  | | 14271 | |  | | 1516 | |  | | 0 | |
| 11417 |  | Val149Phe |  | Val149Leu |  |  |  | nsp6 |  | G |  | 0 |  | | 15751 | |  | | 36 | |  | | 2 | |
| 12079 |  | Syn |  | Syn |  |  |  | nsp7 |  | G |  | 9 |  | | 15779 | |  | | 0 | |  | | 1 | |
| 12741 |  | Thr19Ile |  | Thr19Lys |  |  |  | nsp9 |  | C |  | 2 |  | | 0 | |  | | 8 | |  | | 15779 | |
| 14118 |  | Syn |  | Syn |  |  |  | nsp12 |  | G |  | 3 |  | | 15776 | |  | | 10 | |  | | 0 | |
| 15354 |  | Syn |  | Syn |  |  |  | nsp12 |  | T |  | 1 |  | | 0 | |  | | 15761 | |  | | 27 | |
| 16272 |  | Syn |  | Syn |  |  |  | nsp13 |  | T |  | 0 |  | | 27 | |  | | 15761 | |  | | 1 | |
| 17010 |  | Syn |  | Syn |  |  |  | nsp13 |  | C |  | 7 |  | | 0 | |  | | 29 | |  | | 15753 | |
| 17410 |  | Arg392Met |  | Arg392Cys |  |  |  | nsp13 |  | C |  | 6 |  | | 0 | |  | | 46 | |  | | 15737 | |
| 18756 |  | Syn |  | Syn |  |  |  | nsp14 |  | G |  | 2 |  | | 15774 | |  | | 13 | |  | | 0 | |
| 19170 |  | Syn |  | Leu |  |  |  | nsp14 |  | C |  | 1 |  | | 0 | |  | | 83 | |  | | 15705 | |
| 20134 |  | Val172Leu |  | Val172Ile |  |  |  | nsp15 |  | G |  | 1 |  | | 15743 | |  | | 45 | |  | | 0 | |
| 20275 |  | Asp219Asn |  | Asp219Tyr |  |  |  | nsp15 |  | G |  | 24 |  | | 15760 | |  | | 5 | |  | | 0 | |
| 21452 |  | Gly265Val |  | Gly265Asp |  |  |  | nsp16 |  | G |  | 1 |  | | 15758 | |  | | 30 | |  | | 0 | |
| 21724 |  | Leu54Phe |  | Syn |  |  |  | SPIKE |  | G |  | 0 |  | | 15762 | |  | | 26 | |  | | 1 | |
| 21974 |  | Asp138His |  | Asp138Tyr |  |  |  | SPIKE |  | G |  | 0 |  | | 15769 | |  | | 3 | |  | | 17 | |
| 23422 |  | Syn |  | Syn |  |  |  | SPIKE |  | C |  | 1 |  | | 0 | |  | | 15 | |  | | 15773 | |
|  |  |  |  |  |  |  |  |  |  |  |  |  |  | |  | |  | |  | |  | |  | |
| **Table S1** (cont.) | | | | | | | | | | | | | | | | | | | | | | | | |
| **Site** |  | **Mut01** |  | **Mut02** |  | **Mut03** |  | **Gene** |  | **Ref** |  | **A** |  | | **G** | |  | | **T** | |  | | **C** | |
| 23587 |  | Gln675His |  | Syn |  |  |  | SPIKE |  | G |  | 0 |  | | 15769 | |  | | 3 | |  | | 17 | |
| 23856 |  | Arg765His |  | Arg765Thr |  |  |  | SPIKE |  | G |  | 6 |  | | 15776 | |  | | 7 | |  | | 0 | |
| 24197 |  | Ala879Ser |  | Ala879Thr |  |  |  | SPIKE |  | G |  | 3 |  | | 15753 | |  | | 33 | |  | | 0 | |
| 24368 |  | Asp936Tyr |  | Syn |  |  |  | SPIKE |  | G |  | 0 |  | | 15660 | |  | | 128 | |  | | 1 | |
| 25429 |  | Val13Leu |  | Val13Ile |  |  |  | ORF3A |  | G |  | 1 |  | 15581 | |  | | 207 | |  | | 0 | |  |
| 25463 |  | Thre24Ile |  | Thre24Asn |  |  |  | ORF3A |  | C |  | 2 |  | 0 | |  | | 8 | |  | | 15779 | |  |
| 25500 |  | Syn |  | Syn |  |  |  | ORF3A |  | G |  | 2 |  | 15764 | |  | | 23 | |  | | 0 | |  |
| 25505 |  | Gln38Pro |  | Gln38Leu |  |  |  | ORF3A |  | A |  | 15772 |  | 0 | |  | | 2 | |  | | 15 | |  |
| 25521 |  | Syn |  | Leu |  |  |  | ORF3A |  | C |  | 2 |  | 0 | |  | | 13 | |  | | 15774 | |  |
| 25563 |  | Gln57His |  | Syn |  |  |  | ORF3A |  | G |  | 0 |  | 11044 | |  | | 4743 | |  | | 2 | |  |
| 25593 |  | Lys67Asn |  | Syn |  |  |  | ORF3A |  | G |  | 0 |  | 15772 | |  | | 6 | |  | | 11 | |  |
| 25688 |  | Ala99Asp |  | Ala99Val |  |  |  | ORF3A |  | C |  | 10 |  | 0 | |  | | 71 | |  | | 15708 | |  |
| 26152 |  | Gly254Arg |  | Gly254STOP |  |  |  | ORF3A |  | G |  | 10 |  | 15777 | |  | | 2 | |  | | 0 | |  |
| 26730 |  | Val70Ile |  | Syn |  |  |  | MEM |  | G |  | 9 |  | 15761 | |  | | 19 | |  | | 0 | |  |
| 27327 |  | Lys42Asn |  | Syn |  |  |  | ORF6 |  | G |  | 1 |  | 15776 | |  | | 12 | |  | | 0 | |  |
| 27669 |  | Syn |  | Lys92Asp |  |  |  | ORF7A |  | A |  | 15768 |  | 20 | |  | | 1 | |  | | 0 | |  |
| 28077 |  | Val62Leu |  | Syn |  |  |  | ORF8 |  | G |  | 0 |  | 15568 | |  | | 45 | |  | | 176 | |  |
| 28311 |  | Pro13Leu |  | Pro13Arg |  |  |  | NUC |  | C |  | 0 |  | 4 | |  | | 250 | |  | | 15535 | |  |
| 28344 |  | Thr24Asn |  | Thr24Ile |  |  |  | NUC |  | C |  | 23 |  | 0 | |  | | 1 | |  | | 15765 | |  |
| 28378 |  | Ala35Ser |  | Syn |  |  |  | NUC |  | G |  | 1 |  | 15768 | |  | | 19 | |  | | 1 | |  |
| 28580 |  | Asp103Tyr |  | Syn |  |  |  | NUC |  | G |  | 4 |  | 15725 | |  | | 60 | |  | | 0 | |  |
| 28705 |  | Syn |  | Syn |  |  |  | NUC |  | T |  | 0 |  | 1 | |  | | 15776 | |  | | 12 | |  |
| 28849 |  | Syn |  | Syn |  |  |  | NUC |  | C |  | 0 |  | 2 | |  | | 14 | |  | | 15773 | |  |
| 28851 |  | Ser193Ile |  | Ser193Thr |  |  |  | NUC |  | G |  | 0 |  | 15712 | |  | | 71 | |  | | 6 | |  |
| 28857 |  | Arg195Lys |  | Syn |  |  |  | NUC |  | G |  | 12 |  | 15775 | |  | | 2 | |  | | 0 | |  |
| 28878 |  | Ser202Asn |  | Ser202Thr |  |  |  | NUC |  | G |  | 118 |  | 15665 | |  | | 2 | |  | | 4 | |  |
| 28881 |  | Arg203Lys |  | Arg203Met |  |  |  | NUC |  | G |  | 3300 |  | 12485 | |  | | 4 | |  | | 0 | |  |
| 28882 |  | Syn |  | Syn |  |  |  | NUC |  | G |  | 3293 |  | 12493 | |  | | 3 | |  | | 0 | |  |
| 28883 |  | Gly204Arg |  | Syn |  |  |  | NUC |  | G |  | 1 |  | 12496 | |  | | 0 | |  | | 3292 | |  |
| 28899 |  | Arg209Lys |  | Arg209Ile |  | Arg209Thr |  | NUC |  | G |  | 2 |  | 15773 | |  | | 5 | |  | | 9 | |  |
| 29540 |  | Non-coding |  | Non-coding |  |  |  | NC |  | G |  | 205 |  | 15576 | |  | | 8 | |  | | 0 | |  |
| 29543 |  | Non-coding |  | Non-coding |  |  |  | NC |  | G |  | 0 |  | 15763 | |  | | 9 | |  | | 17 | |  |
| 29567 |  | Ile4Leu |  | Ile4Val |  |  |  | ORF10 |  | A |  | 15776 |  | 1 | |  | | 0 | |  | | 12 | |  |

**Table S2.**

Groups of conservative substitutions considered in this study. Amino acids in brackets are evaluated after structural analysis.

| **Group** | **Amino acid** |
| --- | --- |
| Aromatic | Y, W, [H], [F] |
| Non-polar | A, I, V, L, M, [F], [P] |
| Polar, hydrogen bond interacting | S, T, [C] |
| Glycine | G |
| Amidic, hydrogen bond interacting | N, Q |
| Acidic | D, E |
| Basic | R, H, K |

**Table S3.**

Results from iRF-LOOP.  Significant loci based on their ability to predict or be predicted by other loci.  Three sites (21575_T, 11083_T, and 11074_T) appear to be hypermutation sites that occur as the result of long stretches of thymines due to consecutive runs of phenylalanine, leucine, and valine residues.

| **Site** | **RIT_Score** | **Gene** | **Change** | **Possible Mechanism or Repeat** |
| --- | --- | --- | --- | --- |
| 21575_T | 15992 | S | Phe^5^Leu | Run of Ts from consecutive Phe-Leu |
| 24368_T | 14445 | S | Asp^936^Tyr | Three alleles |
| 19484_T | 12647 | nsp14 | Ala^482^Val | GGT GGT |
| 16887_T | 11650 | nsp13 | Syn | CAA CAA CAA |
| 21137_G | 8979 | nsp16 | Lys^160^Met | GGGTTT GGGTTT |
| 11083_T | 7629 | nsp6 | Syn | Run of Ts from consecutive Phe-Leu |
| 27384_C | 5464 | ORF6 | Syn | GATT GATT |
| 28887_T | 4950 | N | Ser^202^Asn | CAG CAG CAG |
| 11074_T | 4875 | nsp6 | Syn | Run of Ts from consecutive Phe |
| 10376_T | 3925 | nsp5 | Pro^108^Ser | TAAGT TAAGT |
| 10369_T | 3680 | nsp5 | Syn | TAAGT TAAGT |
| 313_T | 2656 | nsp1 | Syn | GTTT GTTT |
| 15324_T | 2625 | nsp12 | Syn | GCC GCC |
| 23311_T | 2613 | S | Glu^583^Asp | CA CA CA CA |
| 26681_T | 2289 | M | Syn | TAA TAA TTTT |
| 20148_T | 1971 | nsp15 | Syn | ATT ATT |
| 29095_T | 1911 | N | Syn | CAA CAA |
| 9438_T | 1888 | nsp4 | Thr^295^Ile | GTA GTA GTA |
| 10323_G | 1715 | nsp5 | Lys^90^Arg | TTAAG TTAAG |

**Table S4.**

Summary of the main mutations, possible observable characteristics they may affect, and suggested future experiments to assess that.

| **Protein** | **Mutations** | **Phenotype** | **Suggested Experiments** |
| --- | --- | --- | --- |
| **Nsp2** | Pro^91^Ser  Thr^85^Ile | Binding affinity to ORF3a and PHB | Co-immunoprecipitation [[2]](https://paperpile.com/c/2O3uyJ/twWIK) |
| **Nsp3** | Thr^1198^Lys  Thr^1198^Ile  Thr^1198^Arg | Binding affinity to RNA | Electrophoretic mobility assays [3] |
| **Nsp7** | Ser2^5^Leu | Binding to nsp8 in the replicase complex; replication efficiency | Small-angle neutron and X-ray scattering;  Biochemical assays |
| **Nsp12** | Pro3^23^Leu | Local flexibility; binding to nsp8 in the replicase complex; replication efficiency | Small-angle neutron and X-ray scattering;  Biochemical assays |
| **Nsp13** | Pro^504^Leu  Tyr^541^Cys | Helicase efficiency | Nucleic acid unwinding and ATP hydrolysis assays [4] |
| **N** | Ser^188^Leu  Ser^193^Ile  Ser^195^Leu  Ser^197^Leu  Ser^202^Asn(Ile)  Thr^205^Ile  Arg^203^Lys  Gly^204^Arg  (Arg^203^Lys + Gly^204^Arg) | Oligomerization; binding to nsp3; replication performance of the virus | Two-hybrid assays [5] |
| **S** | Asp^614^Gly | Trimer packing; infectivity | Cryo-EM |

**Supplementary References**

1. Jia Z, Yan L, Ren Z, Wu L, Wang J, Guo J, et al. Delicate structural coordination of the Severe Acute Respiratory Syndrome coronavirus Nsp13 upon ATP hydrolysis. Nucleic Acids Res. 2019;47:6538–50.

2. von Brunn A, Teepe C, Simpson JC, Pepperkok R, Friedel CC, Zimmer R, et al. Analysis of intraviral protein-protein interactions of the SARS coronavirus ORFeome. PLoS One. 2007;2:e459.

3. Neuman BW, Joseph JS, Saikatendu KS, Serrano P, Chatterjee A, Johnson MA, et al. Proteomics analysis unravels the functional repertoire of coronavirus nonstructural protein 3. J Virol. 2008;82:5279–94.

4. Jia Z, Yan L, Ren Z, Wu L, Wang J, Guo J, et al. Delicate structural coordination of the Severe Acute Respiratory Syndrome coronavirus Nsp13 upon ATP hydrolysis. Nucleic Acids Res. 2019;47:6538–50.

5. He R, Dobie F, Ballantine M, Leeson A, Li Y, Bastien N, et al. Analysis of multimerization of the SARS coronavirus nucleocapsid protein. Biochem Biophys Res Commun. 2004;316:476–83.
