# Supplementary material for: Potentially adaptive SARS-CoV-2 mutations discovered with novel spatiotemporal and explainable AI models
Source: Genome Biol. 2020 Dec 23;21:304. doi: 10.1186/s13059-020-02191-0 (PMC7756312; doi:10.1186/s13059-020-02191-0)
Supplement: Supplementary file 3 — Additional file 3. Review history. [file 13059_2020_2191_MOESM3_ESM.docx]

**1^st^ round**

**Reviewer 1**

Review of "Potentially Adaptive SARS-CoV-2 Mutations Discovered with Novel Spatiotemporal and Explainable-AI Models" by Garvin et al. (GBIO-D-20-01238)

In this study, Garvin and colleagues analyzed 15,789 SARS-CoV-2 genomes and explored the possible evolutionary forces driving the spread of different haplotypes. The authors inferred the fitness of genetic variants of SARS-CoV-2 viruses based on several parameters, such as their duration, dispersal, and frequency in the host population. Consistent with several previous studies, the authors also reported a handful of mutations that are tightly linked across these viral genomes. The authors argued that the linkage of the mutations could be caused by compensatory adaptive changes. In particular, the authors predicted the D614G mutation in the S protein would reduce transmission, and the subsequent P323L mutation in RDRP compensated the deleterious effect of the D614G.

Overall this manuscript is well written. The questions the authors have been addressing are definitely crucial. Nevertheless, the analysis performed in this manuscript is not yet adequate to support the conclusions, and the present form of the manuscript is still speculative. Additional studies are needed to provide further evidence for the claims.

Specific comments:

1). In the "gene-based analysis" part (lines 141-), the authors conducted dN/dS analysis to detect the signature of positive selection on individual genes. Although dN/dS > 1 is indicative of positive selection, such a result can also be obtained due to purifying selection on dS but not necessarily elevated dN. The authors should provide evidence that synonymous mutations are strictly neutral in the SARS-Cov-2 genomes.

2) It is not clear whether the dN/dS > 1 is statistically significant. Since, on average, ~8-10 mutations exist between a SARS-CoV-2 genome and the Hu-1 reference genome, stochastical errors would cause large variation of the dN/dS values. Also, the authors should incorporate all the sequences in the analysis, and conducted pairwise comparisons, rather than arbitrarily selecting the genomes are divergent from the reference genomes. Moreover, it is not clear how the authors obtained the dN/dS values. The detailed procedures should be given. Furthermore, the authors can consider using more reasonable (and sophisticated) algorithms such as codeml in the PAML package to detect the signatures of positive selection.

3) It is not clear how the wavelet analysis was modeled and conducted. The authors should give more detailed on the biological reasoning 1) why the wavelet analysis can be applied here, 2) what does the y-axis mean, and 3) why the peaks of the y-axis inform natural selection.

4) In the "Haplotype success and potential adaptation" part (Line206-), the authors defined the "successful" haplotypes by considering the temporal and spatial distributions of the haplotypes. One caveat in the analysis is the authors did not consider the bias in sample collection and sequencing in the GISAID database. For example, a very large fraction of the SARS-CoV-2 genomes deposited in GISAID was sequenced in UK. And many haplotypes might exist but were not sequenced and covered in this study. How would the sampling bias affect the conclusion here?

5) In Figure 2, the authors presented the haplotype networks of the viral genomes. And in Figure 3, the authors summarized different types of mutations. The assumption of these analyses is that the reference genome (Hu-1) is the ancestor, and all the other sequences are derived from the Hu-1 genome. A similar logic was also adopted in other parts of this study. This is not correct. Previous studies such as Forster's PNAS and Tang's NSR have already demonstrated that RaTG13 should be used to polarize the mutations in the SARS-CoV-2 populations.

6) The authors argued that D614G mutation was followed and rescued by the P323L in the nsp12. Again, this conclusion should be examined with RaTG13 as the outgroup. Also, the possible compensatory adaptive events in other locations of nsp12 and nsp13 (Lines 256-289) should be re-analyzed using an outgroup as well.

7) In the analysis of "Multiple mutations at a site", the authors should cite the previous studies such as Rice et al. (PMID: 32687176), who have carried out a more detailed investigation on this topic.

8) The whole section of "Structural analysis of SARS-CoV-2 mutants" is interesting, but speculative and boring. Can the authors provide any experimental evidence that the inferred adaptive variants are indeed biological functional?

**Reviewer 2**

Main Findings:

The main findings of this paper are a detailed structural mutational analysis, done geo-temporally (which is very valuable), and exhaustively overlaid with analysis of structural mutations across the genome. The analysis provides unique insight that will be complementary (and somewhat contrary) to mutational analyses that are currently known on the viral genome. A main point in this paper is the assertion that the well-known Asp614Gly mutation is not necessarily the causative mutation for infectivity, but that a compensatory mutation in the nsp12 may be responsible. The extensive structural analysis are important and will be useful for hypothesis generation (of which the author did a lot of), and testing whether certain mutations can be geographically important for persistence and targeted for therapeutics.

**Author response**

Reviewer 1:

In this study, Garvin and colleagues analyzed 15,789 SARS-CoV-2 genomes and explored the possible evolutionary forces driving the spread of different haplotypes. The authors inferred the fitness of genetic variants of SARS-CoV-2 viruses based on several parameters, such as their duration, dispersal, and frequency in the host population. Consistent with several previous studies, the authors also reported a handful of mutations that are tightly linked across these viral genomes. The authors argued that the linkage of the mutations could be caused by compensatory adaptive changes. In particular, the authors predicted the D614G mutation in the S protein would reduce transmission, and the subsequent P323L mutation in RDRP compensated the deleterious effect of the D614G.

Overall this manuscript is well written. The questions the authors have been addressing are definitely crucial. Nevertheless, the analysis performed in this manuscript is not yet adequate to support the conclusions, and the present form of the manuscript is still speculative. Additional studies are needed to provide further evidence for the claims.

We thank the reviewer for the positive comments and questions raised.

Specific comments:

1). In the "gene-based analysis" part (lines 141-), the authors conducted dN/dS analysis to detect the signature of positive selection on individual genes. Although dN/dS > 1 is indicative of positive selection, such a result can also be obtained due to purifying selection on dS but not necessarily elevated dN. The authors should provide evidence that synonymous mutations are strictly neutral in the SARS-Cov-2 genomes.

The Systems Biology approach used in this study measures selection with different models values indicate that several proteins may be under positive selection with the highest signal in nsp2 (Fig. 1A).” (page 8, lines 153-155)

In addition, later in the manuscript we point out that the synonymous change in nsp3 may in fact represent a signature of positive selection via codon bias (page 12, lines 249-257). That is, under that model, it is possible that a synonymous mutation is not neutrally evolving.

2) It is not clear whether the dN/dS > 1 is statistically significant. Since, on average, ~8-10 mutations exist between a SARS-CoV-2 genome and the Hu-1 reference genome, stochastical errors would cause large variation of the dN/dS values. Also, the authors should incorporate all the sequences in the analysis, and conducted pairwise comparisons, rather than arbitrarily selecting the genomes are divergent from the reference genomes. Moreover, it is not clear how the authors obtained the dN/dS values. The detailed procedures should be given. Furthermore, the authors can consider using more reasonable (and sophisticated) algorithms such as codeml in the PAML package to detect the signatures of positive selection.

We provide the dN/dS analysis, which is probably the most widely used measure of molecular evolution, but we simultaneously identify the assumptions and limitations of this metric. The dN/dS values are computed based on the 385 haplotypes that we have detected amongst the 15,789 sequences analyzed (please see the detailed procedure in the updated Methods section, page 32, lines 700-719). A statistical test would require either randomly sampling the 15,789 sequences and calculating dN/dS, or randomly sampling the 385 haplotypes present in the network. In the former case, as the reviewer correctly points out, sampling bias will generate unreliable estimates. In the latter case where we used all known haplotypes after removing rare ones, random sampling will remove information and the small sample sizes would make any claims of significance suspect. Instead, we report the dN/dS ratio as one model, with known limitations and proceed to search for signatures of selection using additional models including structural and functional predictions of the mutations. The use of the 385 haplotypes here reduces bias because the estimates of dN/dS are not affected by the frequency of the haplotypes. It is robust as long as there is sufficient sampling of the entire haplotype space (15,789 genomes were used here, which is much larger than previous analyses that we are aware of). We have clarified this in that section of the manuscript (see tracked changes). Furthermore, we did not use rare haplotypes (fewer than five individuals in 15,789) in order to rule out any that may be due to sequencing error. Finally, the reviewer’s suggestion of performing pairwise comparisons would be highly biased by the differences in sampling as they have pointed out in other sections. The use of only the 385 haplotypes present provides an unbiased estimate of dN/dS. If the reviewer’s suggestion was to perform pairwise comparisons (and their associated assumptions) in order to gather multiple lines of evidence that converge on what is most likely to be functionally and evolutionarily important. We agree with the reviewer that dN/dS analysis can be prone to error due to purifying selection on dS and we do not claim that synonymous mutations are evolving neutrally in SARS-CoV-2 genomes. That is the assumption of the dN/dS model. To reinforce that, we have added a parenthetical comment that the model assumes synonymous mutations are neutral.

“Given the assumptions of the dN/dS model (i.e., that synonymous mutations are neutral), the values indicate that several proteins may be under positive selection with the highest signal in nsp2 (Fig. 1A).” (page 8, lines 153-155)

In addition, later in the manuscript we point out that the synonymous change in nsp3 may in fact represent a signature of positive selection via codon bias (page 12, lines 249-257). That is, under that model, it is possible that a synonymous mutation is not neutrally evolving.

2) It is not clear whether the dN/dS > 1 is statistically significant. Since, on average, ~8-10 mutations exist between a SARS-CoV-2 genome and the Hu-1 reference genome, stochastical errors would cause large variation of the dN/dS values. Also, the authors should incorporate all the sequences in the analysis, and conducted pairwise comparisons, rather than arbitrarily selecting the genomes are divergent from the reference genomes. Moreover, it is not clear how the authors obtained the dN/dS values. The detailed procedures should be given. Furthermore, the authors can consider using more reasonable (and sophisticated) algorithms such as codeml in the PAML package to detect the signatures of positive selection.

We provide the dN/dS analysis, which is probably the most widely used measure of molecular evolution, but we simultaneously identify the assumptions and limitations of this metric. The dN/dS values are computed based on the 385 haplotypes that we have detected amongst the 15,789 sequences analyzed (please see the detailed procedure in the updated Methods section, page 32, lines 700-719). A statistical test would require either randomly sampling the 15,789 sequences and calculating dN/dS, or randomly sampling the 385 haplotypes present in the network. In the former case, as the reviewer correctly points out, sampling bias will generate unreliable estimates. In the latter case where we used all known haplotypes after removing rare ones, random sampling will remove information and the small sample sizes would make any claims of significance suspect. Instead, we report the dN/dS ratio as one model, with known limitations and proceed to search for signatures of selection using additional models including structural and functional predictions of the mutations. The use of the 385 haplotypes here reduces bias because the estimates of dN/dS are not affected by the frequency of the haplotypes. It is robust as long as there is sufficient sampling of the entire haplotype space (15,789 genomes were used here, which is much larger than previous analyses that we are aware of). We have clarified this in that section of the manuscript (see tracked changes). Furthermore, we did not use rare haplotypes (fewer than five individuals in 15,789) in order to rule out any that may be due to sequencing error. Finally, the reviewer’s suggestion of performing pairwise comparisons would be highly biased by the differences in sampling as they have pointed out in other sections. The use of only the 385 haplotypes present provides an unbiased estimate of dN/dS. If the reviewer’s suggestion was to perform pairwise comparisons within the 385 haplotypes, we note that nearly every edge in the network is a single mutational event. A pairwise comparison will therefore simply reproduce what we already have.

As we pointed out in the manuscript:

”Detection of signatures of selection with the dN/dS analysis is an especially robust and effective approach when comparing sequences that represent significant time intervals since divergence (e.g. sequence variation at species-level). However, the models developed on this metric are built around fixed mutations in each species and not segregating polymorphisms within a species (i.e. population-level) and, therefore it can miss selective events [52].” (page 8, lines 157-161)

The reviewer’s suggestion that we use models available in the codeml/PAML package is addressed in the above quote from the manuscript. This quote is a caveat of dN/dS analysis in general, but it is especially applicable to the suggested use of codeml/PAM in this study. That is, various models within codeml/PAML use dN/dS estimates to identify signatures of selection and are robust at the species level but rarely at the within species population level. Indeed, Ziheng Yang’s lab, which developed PAML, states that the site-directed evolutionary analysis that the algorithm performs is conservative and is unlikely to detect selection on short evolutionary time scales (Yang 2006). The reviewer’s concern may be referring to phylogenetic analyses of large evolutionary timescales in which dN/dS ratios are estimated. Here we can actually count them because our evolutionary scale is in days. There is no need to estimate the mutation rate as we are observing it in real time. Yang’s lab does point out that PAML may be able to identify signatures of selection for viruses like HIV (Yang 2006). However, that is because HIV has a substantially higher mutation rate compared to coronaviruses. (Yang Z. Computational Molecular Evolution [Internet]. 2006. Available from: <http://dx.doi.org/10.1093/acprof:oso/9780198567028.001.0001>)

3) It is not clear how the wavelet analysis was modeled and conducted. The authors should give more detailed on the biological reasoning 1) why the wavelet analysis can be applied here, 2) what does the y-axis mean, and 3) why the peaks of the y-axis inform natural selection.

(1) With the Systems Biology approach used here, we search for measures of selection and mutation distributions using different models aiming to weaken errors due to their limitations and converge on the most important evolutionary processes. The wavelet analysis is able to simultaneously represent the density, frequency, and location of mutations across the genome. We have added text in that section to better clarify this (page 9, lines 169-170).

(2) The y-axis corresponds to the density of the wavelet across the genome as a log-scale (this is defined in the figure legend). We have adjusted the y-axis label in the Figure.

(3) The major signal here that informs natural selection is the “functional homoplasy” at the nucleocapsid gene that we discuss in the text.

4) In the "Haplotype success and potential adaptation" part (Line206-), the authors defined the "successful" haplotypes by considering the temporal and spatial distributions of the haplotypes.

One caveat in the analysis is the authors did not consider the bias in sample collection and sequencing in the GISAID database. For example, a very large fraction of the SARS-CoV-2 genomes deposited in GISAID was sequenced in UK. And many haplotypes might exist but were not sequenced and covered in this study. How would the sampling bias affect the conclusion here?

We agree that using the GISAID database can lead to sampling bias. Because of that, our focus is on those haplotypes that are not successful but become so after a mutational event - oversampling will therefore have little effect on this metric. As stated in the manuscript: “The most successful haplotypes under this model are those that persist for an extended period of time before mutating and they also infect large numbers of individuals across many geographic regions. Particularly, we are very interested in viral types that are unsuccessful according to our model but acquire improved fitness with a subsequent mutational event as these represent potentially adaptive or compensatory responses. These ineffective haplotypes will be those that persist for many days and are found in many geographic regions but at low frequency. ” (page 11, lines 226-232)

We updated the Methods section to reflect this: “The division by the number of geographic regions in which the haplotype was found and by using coarse definitions of geography (i.e. continents rather than countries) we reduce sampling bias that is known to occur in GISAID.” Bias due to oversampling of sequences in one location (e.g., the UK) is minimized because, in the metric applied (pages 11-15, lines 216-297), the high success also requires sampling across other geographic regions and large timespans. Furthermore we use 6 broad geographic regions in our metric (Africa, Asia, Europe, North America, Oceana, and South America, see Figure 2) and therefore bias is reduced because local sampling effects end up being distributed across a continent (e.g., the sample numbers for the UK are distributed across Europe). In fact, the genomic space that we have analyzed here is well sampled because the majority of the edges in our haplotype networks represent single mutations. Therefore, any low-sampled haplotypes are either those that exist in GISAID (and therefore are included in our network), or they are terminal nodes, which are not important for our success metric (as we state in the manuscript, our interest is in internal nodes).

5) In Figure 2, the authors presented the haplotype networks of the viral genomes. And in Figure 3, the authors summarized different types of mutations. The assumption of these analyses is that the reference genome (Hu-1) is the ancestor, and all the other sequences are derived from the Hu-1 genome. A similar logic was also adopted in other parts of this study. This is not correct. Previous studies such as Forster's PNAS and Tang's NSR have already demonstrated that RaTG13 should be used to polarize the mutations in the SARS-CoV-2 populations.

Use of the RaTG13 sequence as reference is not appropriate for the goals of this study. Tang et al 2020, “On the origin and continuing evolution of SARS-CoV-2” National Science Review 7: 1012-1023, is focused on the origin of the SARS-CoV-2 virus and on the comparison with other species of betacoronavirus. In contrast, our focus here is on the mutational record of the SARS-CoV-2 outbreak, defining Wuhan (December 2019) as the starting point of the spread to the remainder of the human population. Additionally, we stress that we have used more than 15,000 sequences, in contrast, Tang et al only use 103 sequences; any frequency estimates of their work were, therefore, likely to be highly biased. We advocate that the inclusion of RaTG13 or other species of coronavirus is not only inappropriate but it will, at the very least, introduce confusion. Most importantly, it would not change the relationships of the haplotypes we report here. Comparison of the network we generated from fewer sequences in an earlier version of this manuscript emphasizes the robustness of our results (Fig. S8).

6) The authors argued that D614G mutation was followed and rescued by the P323L in the nsp12. Again, this conclusion should be examined with RaTG13 as the outgroup. Also, the possible compensatory adaptive events in other locations of nsp12 and nsp13 (Lines 256-289) should be re-analyzed using an outgroup as well.

The success metric that we use in our analysis is based on the sampling date of each haplotype. In order to re-analyze the adaptive events with an outgroup we would need a sampling date for the RaTG13 virus - specifically when it first appeared in nature. This is not known and any estimate would be based on a sequence analysis which would confound any downstream analyses. Furthermore, as we point out above, this is not a traditional phylogenetic analysis meant to place the SARS-CoV-2 virus in the context of other coronaviruses. Rather, we are modeling the first 68 days of the mutation record to identify potential signatures of positive (and purifying) selection. Inclusion of the RaTG13 sequence therefore is not appropriate in this context and would introduce substantial error in any estimates. Finally, as we point out in the Methods section, inclusion of ancestral sequences to the Wuhan reference will not change the overall topology of the network we have generated (pages 32, lines 687-693).

7) In the analysis of "Multiple mutations at a site", the authors should cite the previous studies such as Rice et al. (PMID: 32687176), who have carried out a more detailed investigation on this topic.

Thank you for pointing out this reference. We have now cited this paper.

8) The whole section of "Structural analysis of SARS-CoV-2 mutants" is interesting, but speculative and boring. Can the authors provide any experimental evidence that the inferred adaptive variants are indeed biological functional?

It is unfortunate that the reviewer finds this section boring. As this is a personal opinion, we do not see how to address that. Like ourselves, we believe other readers will enjoy it.

For example, Reviewer #2 stated:

“The extensive structural analyses are important and will be useful for hypothesis generation (of which the author did a lot of), and testing whether certain mutations can be geographically important for persistence and targeted for therapeutics.”

We do in fact provide experimental and computational evidence indicative that these mutations are biologically relevant in several instances. The hypotheses we raised when discussing all the main targets of mutation are strengthened with reported experimental results from SARS-CoV-2 or, in some cases, SARS-CoV studies. For example, for the Pro323Leu mutation in nsp12, we use the mutation rate in conjunction with the haplotype network to demonstrate greater numbers of mutations in individuals that carry this substitution. This supports previous experimental work of Pachetti et al. 2020 (page 19-20, lines 401-415). We also cite Tylor et al 2009, who performed in vitro mutational analyses at residues in the Ser/Arg-rich region of the nucleocapsid in the related SARS-CoV virus that negatively affected its ability to replicate (page 22, lines 468- 479).

However, in contrast to other studies that focus on individual mutations, the present study highlights the importance of considering concurrent mutations to evaluate their impact in viral transmissibility. Thus, we aim to provide a global picture of mutational events and, with that, point out directions for a collection of future experiments and computational studies. Given the challenges of each system, different expertise will be needed for experimental follow-up. It would be much less timely for us to pursue experimental results that will likely take many months for our group to do on our own. In the spirit of a united research community rising to meet the challenges posed by this pandemic, this study serves exactly the purpose of mobilizing diverse expertise worldwide. We have added sentences in the Concluding Remarks section to reflect this aim (pages 30-31) as well as a supplementary Table (Table S6) that summarizes the highlighted mutations and the suggested experiments to address the hypothesis raised in this study.

Reviewer #2: Main Findings:

The main findings of this paper are a detailed structural mutational analysis, done geotemporally (which is very valuable), and exhaustively overlaid with analysis of structural mutations across the genome. The analysis provides unique insight that will be complementary (and somewhat contrary) to mutational analyses that are currently known on the viral genome. A main point in this paper is the assertion that the well-known Asp614Gly mutation is not necessarily the causative mutation for infectivity, but that a compensatory mutation in the nsp12 may be responsible. The extensive structural analysis are important and will be useful for hypothesis generation (of which the author did a lot of), and testing whether certain mutations can be geographically important for persistence and targeted for therapeutics.

We would like to thank Reviewer 2 for their thorough reading of the manuscript.

**2^nd^ round**

**Reviewer 1**

Review of " Potentially Adaptive SARS-CoV-2 Mutations Discovered with Novel Spatiotemporal and Explainable-AI Models " by Garvin et al. (GBIO-D-20-01238R1).

The authors have addressed most of my concerns in this revised version. However, I still have two concerns which might affect the major conclusions of this study.

1. The authors argued that "Use of the RaTG13 sequence as reference is not appropriate for the goals of this study". I don't agree with this point and the related analysis. The haplotype networks should be correctly rooted to reflect the evolutioary trajectory of the viruses. The authors defined Wuhan (December 2019) and the reference genome (Wu-Hu-1) as the starting point of the spread to the remainder of the human population. That is not correct. The Wu-Hu-1 was among the first viral strains to be isolated, but that does not necessarily suggest that all the viruses currently circulating are the direct descendants of that strain. It is possible that other older strains were not sequenced but transmitted. This issue should be addressed.

2. The authors suggested that the D614G mutation in the Spike protein likely reduced transmission of SARS-CoV-2, and the authors attributed the molecular mechanism to stabilizing effect of the D614G mutation to the S trimer, based on molecular dynamic simulations analyses. This conclusion is in sharp contrast to the finding of a recent Cell paper (https://doi.org/10.1016/j.cell.2020.09.032), which characterized the D614G mutant using cryo-EM and showed that the D614G mutation is likely more infectious, since the mutation shifts the S protein conformation towards an ACE2-binding fusion-competent state. This needs to be reconciled. The authors performed their analyses using a 10 µs simulation of the S protein provided by the D. E. Shaw Institute, which is likely too short to reflect the conformational landscape of the S protein.

**Reviewer 2**

See previous comments; This article is acceptable for publication.

**Author response**

Question 1. The authors argued that "Use of the RaTG13 sequence as reference is not appropriate for the goals of this study". I don't agree with this point and the related analysis. The haplotype networks should be correctly rooted to reflect the evolutionary trajectory of the viruses. The authors defined Wuhan (December 2019) and the reference genome (Wu-Hu-1) as the starting point of the spread to the remainder of the human population. That is not correct. The Wu-Hu-1 was among the first viral strains to be isolated, but that does not necessarily suggest that all the viruses currently circulating are the direct descendants of that strain. It is possible that other older strains were not sequenced but transmitted. This issue should be addressed.

Comment: We agree with Reviewer #1 that the Wu-Hu-1 reference sequence is very likely not the original SARS-CoV-2 variant that infected humans. We repeat here what we said in our initial rebuttal to this point, namely that our interest here is the movement and mutation record of the SARS-CoV-2 virus that is causing the pandemic and appeared after Wu-Hu-1. Therefore, we have used that as the reference sequence. If we were attempting to identify the origin of the virus we would explore other variants such as RaTG13, but that is not what we are doing here. We have updated the methods to specifically state that we used Wu-Hu-1. It is common practice in population genomics to pick one single genotype as a reference to which all others are compared, which is what we have done here.

Question 2. The authors suggested that the D614G mutation in the Spike protein likely reduced transmission of SARS-CoV-2, and the authors attributed the molecular mechanism to stabilizing effect of the D614G mutation to the S trimer, based on molecular dynamic simulations analyses. This conclusion is in sharp contrast to the finding of a recent Cell paper (https://doi.org/10.1016/j.cell.2020.09.032), which characterized the D614G mutant using cryo- EM and showed that the D614G mutation is likely more infectious, since the mutation shifts the S protein conformation towards an ACE2-binding fusion-competent state. This needs to be reconciled. The authors performed their analyses using a 10 μs simulation of the S protein provided by the D. E. Shaw Institute, which is likely too short to reflect the conformational landscape of the S protein.

Comment: The recent Cell paper is, in fact, very relevant and the reported results have now been incorporated into our discussion (Yurkovetskiy et al., 2020). We thank the reviewer for pointing this paper out. However, this paper actually supports our hypothesis that Asp614Gly S may lead to an increased propensity to extreme symptoms due to higher infectivity, which are only attenuated in combination with Pro323Leu nsp12. As we state in the manuscript, the mutation in S is mostly co-occurring with Pro323Leu in nsp12, which may impair not only proofreading capability but replicase activity. It is important to differentiate between the increased infectivity of Asp614Gly S variant observed in cell culture and the transmission of the virus carrying both the Asp614Gly S and Pro323Leu nsp12 mutations on a population scale. The Cell paper also mentions but does not address the other mutations that are linked to S (one of which is Pro323Leu in nsp12). Essentially, we are pointing out that the effect of the mutation in the nsp12 has not been taken into account when evaluating the spread of the Asp614Gly S variant. In order to make this clearer, we added a paragraph to the manuscript in which we discuss three hypotheses that may explain the prevailing Asp614Gly S - Pro323Leu nsp12 variant.

Depending on the accuracy of the initial structure used, molecular dynamics simulations at the microsecond timescale can well be used to describe local flexibility and wider motions of groups of residues (Henzler-Wildman & Kern, 2007) such as loops and ɑ-helices, thus it samples an ensemble of conformations that represents the native state of a protein (Henzler-Wildman & Kern, 2007). That said, although sampling is likely not an issue to obtain local interaction statistics around the 614 residue of S, we opted to remove this analysis for different reasons. These simulations were conducted to analyze the interactions involving Asp614 primarily because the experimentally determined structure available at that time was missing the loops near this site (Walls et al., 2020), which were then modeled in silico. However, this cryo-EM structure, used as starting conformation for the simulations, was generated from a soluble construct of S, which includes two proline stabilizing mutations (Walls et al., 2020) . Very recently, the structure of the full-length S was determined (including those missing loops) and revealed differing structural features in the region near site 614 from the previous structures, likely due to the two proline mutations (Cai et al., 2020). Therefore, we updated our analyses and discussion using the fulllength S structure as reference (PDB id 6xr8) instead of the simulations provided by the D. E. Shaw Institute. Particularly, pairs of interacting residues involving (directly or indirectly) Asp614 were corrected based on the new structure. As we previously described, Asp614 is part of a network of hydrogen bond and salt bridge interactions involving neighboring S protomers, suggesting that this region is a structural relay communicating between the change in residue 614 and the major functional motions of the receptor-binding domains. The updates are found on page 20 (paragraphs 2 and 3), where we also refer to the results from the Cell paper (Yurkovetskiy et al., 2020).

We highlight that the mechanism by which the mutation in 614 leads to higher infectivity is not yet clarified and we do not claim to unravel that. As a multidomain allosteric protein, the action of S is particularly complex. For example, by obtaining the first cryo-EM derived map of the Asp614Gly S variant, Yurkovetskiy et al. suggest that the mutation at residue 614 leads to a major conformational shift toward “open” states, with more than one receptor-binding domain accessible to interact with ACE2. However, despite the higher probability of exposure of the RBDs, the dissociation rate of the S variant to ACE2 is 4-fold faster than the wildtype, showing that the basis of the increased infectivity of Asp614Gly S does not rely on the stronger interaction with ACE2. Later in the manuscript we also state that:

“Therefore, the mechanism by which the mutation in 614 leads to higher infectivity is not fully clear. As it is a multidomain allosteric protein, the involvement of S in cell infection and cell-cell fusion is particularly complex. Further experiments are needed to reconcile the reported higher infectivity, abundance of open states of S, lower affinity to ACE2, and the decreased S1 shedding caused by the Asp614Gly S variant.” (page 20, paragraph 3)

References:

Cai Y, Zhang J, Xiao T, Peng H, Sterling SM, Walsh RM Jr, et al. Distinct conformational states

of SARS-CoV-2 spike protein. Science 369, 1586–92 (2020).

Henzler-Wildman K, Kern D. Dynamic personalities of proteins. Nature 450, 964–972 (2007).

Walls AC, Park Y-J, Tortorici MA, Wall A, McGuire AT, Veesler D. Structure, Function, and

Antigenicity of the SARS-CoV-2 Spike Glycoprotein. Cell 181:281–92.e6 (2020).

Yurkovetskiy L, Wang X, Pascal KE, Tomkins-Tinch C, Nyalile TP, Wang Y, et al. Structural

and Functional Analysis of the D614G SARS-CoV-2 Spike Protein Variant. Cell [Internet]

(2020).
